# A Review of the Design and Synthesis of (Electro)chemically Induced Three-dimensional Nanoporous Copper: A Strategic Element for CO_2_ Electro-Reduction and Self-Sanitization Applications

**DOI:** 10.1021/acsomega.5c08641

**Published:** 2026-02-23

**Authors:** Amirhossein Foroozan Ebrahimy, Yanhong Gu, Irma-Alondra Hermoso-Diaz, Roger C. Newman, Drew Higgins

**Affiliations:** † Corrosion and Advanced Materials Laboratory, Department of Chemical Engineering and Applied Chemistry, 7938 University of Toronto, 200 College Street, Toronto, Ontario M5S 3E5, Canada; ‡ Department of Chemical Engineering, 3710 McMaster University, Hamilton, Ontario L8S 4L7, Canada; § Centro de Investigacion en Ingenieria y Ciencias Aplicadas, Universidad Autonoma del Estado de Morelos, Av. Universidad No.1 1001, Chamilpa, Cuernavaca, Morerlos 62209, Mexico

## Abstract

The catalytic and self-sanitizing properties of copper (Cu) can be significantly enhanced by inducing nanoporosity into the structure. In this review, we first briefly introduce electrocatalysis and biocidal applications of Cu, with the discussion on electrocatalysis geared toward the electrochemical reduction of carbon dioxide to value-added fuels and chemicals. Second, the underlying mechanisms for the enhancement of the electrocatalytic and biocidal properties by means of morphology manipulation are discussed, followed by a review of chemical and electrochemical techniques used to synthesize nanoporous Cu. Additionally, the parameters that enable fine-tuning of the sizes and structures of the resulting porosity are outlined, including the composition and crystal structure of the precursors, along with electrochemical factors such as electrolyte, applied overpotential, and treatment time. This review elucidates the crucial role that the nanostructure of Cu plays in augmenting the electrocatalytic efficiency and self-sterilizing attributes of Cu. Moreover, it provides insights into and discusses the challenges in designing advanced functional nanomaterials and synthesizing well-controlled morphologies essential for sustainable electrocatalysis and antimicrobial applications in diverse fields.

## Graphical abstract

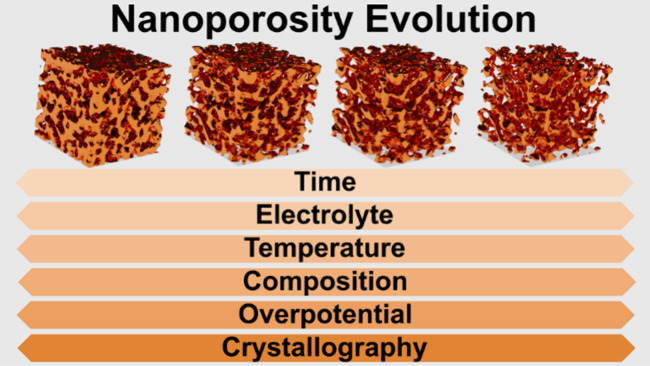

## 1 Introduction

Copper is a native metal; it can be found in its pure metallic form in nature. The so-called copper age, when the utilization of smelted copper became prevalent among human societies, dates to around 5000 years ago. The oldest copper ornaments and tools have been discovered in the Iranian plateau, such as a pendant found in Mesopotamia,^1^ and kitchenware discovered from the Jiroft civilization.^2^ The English noun “copper” originates from the Roman era, when it was mainly mined in Cyprus.^3^ Apart from copper’s well-known applications in wiring, electronics, and heat exchangers related to its high electrical and heat conductivity, copper has garnered tremendous attention as an electrocatalyst and a biocidal material.

Electrocatalysis and biocidal processes are both surface-based phenomena, whereby increasing the surface area to volume ratio of the copper can enhance the performance, owing to the increased availability of active sites. Furthermore, hydrophobicity is often desired in electrocatalysis involving humid gaseous reactants because flooding of catalyst structures with liquid water inhibits the access of reactants to the surface residing active sites. Fine-tuning the morphology and the evolution of hierarchical porous structures^4^ can enable the inducement of superhydrophobicity^5,6^ without the need for the use of toxic per- and polyfluorinated substances (PFAS), also known as forever chemicals. Providing an in-depth understanding of how synthesis parameters influence the final morphology and properties of nanoporous Cu is one of the main goals of this review.

In this article, the introductory remarks around the applications of nanoporous Cu are focused on the electrocatalytic CO_2_ conversion and the self-sanitizing attributes of Cu surfaces. These two applications were selected in view of their strategic importance in facilitating a sustainable, organized human life. The correlation between the increased CO_2_ concentrations in the atmosphere and climate change, with its looming environmental catastrophes and mass migrations, as well as the threat of disease spread and global pandemics, attests to the cruciality of the development of sustainable energy production technologies and self-sterilizing surfaces. Previous reports and reviews have provided a comprehensive assessment of other applications of Cu, which lie outside the scope of this work, including nanomechanics,^7^ interconnections in semiconductors,^8^ sensing,^9,10^ supercapacitors,^11^ batteries,^12^ surface-enhanced Raman spectroscopy,^13^ Fenton-like catalysis,^14^ and catalyzing click reactions in organic synthesis^15^ that interested readers are encouraged to explore.

Moreover, herein we mainly focused on dealloying in aqueous solutions, which is one of the most controllable, facile, and inexpensive methods to synthesize nanoporous Cu, and discussed the role that synthetic parameters play in fine-tuning the resulting porous architecture. Other synthesis methods, which are outside the scope of the present work, include liquid metal dealloying,^16–25^ dealloying in nonaqueous solutions,^26–30^ oxidative–reductive potential pulse treatment,^31–36^ galvanic replacement,^37,38^ conversion reaction synthesis,^39,40^ template-based methods,^41^ magnetron sputtering,^15^ and pulse-periodic LASER treatment.^42–44^

In this review, a brief introduction of the electrocatalytic properties of Cu for electrochemical CO_2_ reduction reaction (CO_2_RR) (Section 1.1) and self-sanitizing effects of Cu surfaces (Section 1.2) is first provided to demonstrate the importance of techniques that would enable the fine-tuning of porosity evolution. Then, chemical and electrochemical techniques, such as dealloying (Section 2) and electrodeposition (Section 3) for the synthesis of standalone, monolithic macroporous (pore diameters >50 nm) and mesoporous (pore diameters between 2 and 50 nm) Cu structures, hereafter generically referred to as nanoporous Cu, are presented. The influence of various synthesis parameters including chemical composition (Section 2.1) and phases and crystal structure (Section 2.2) of the precursors, applied overpotentials (Section 2.3), electrolyte (Section 2.4), temperature (Section 2.5), and treatment duration (Section 2.6) are discussed, such that the quantitative and qualitative adjustments for (electro)­chemical techniques to fine-tune porosity evolution in Cu are elucidated. Lastly, some of the most exciting scientific and engineering opportunities pertaining to the use of nanoporous Cu are highlighted, followed by a discussion of the important challenges and research questions that must be addressed to enable the widespread application of these materials produced by (electro)­chemical techniques (Section 4).

### 1.1 Electrocatalysis Application of Cu toward CO_2_RR

Cu is the only pure transition metal catalyst capable of electrochemically reducing CO_2_ to (oxygenated) hydrocarbons at significant rates.^45–49,45–49,49–57^ The binding energy of the first intermediate during the CO_2_RR, adsorbed carbon monoxide (*CO, where * indicates a surface adsorbed species), is usually used as a descriptor for the electrocatalytic performance of the CO_2_RR on different surfaces. For example, *CO has a relatively weak adsorption (i.e., binding) energy on Au, Ag, and Zn surfaces resulting in its desorption upon CO_2_RR, which explains the high selectivity of Au and Ag catalysts to produce CO.^52^ Catalysts such as Pt and Ni on the other hand have a relatively strong binding energy toward *CO, which commonly results in their surface being poisoned by *CO species which block the catalytically available surface sites and limit the electrocatalytic performance for electrochemical CO_2_RR.^58^ Cu, however, has an intermediate binding strength to *CO that allows for maintaining a balance between the barriers for CO_2_ activation, *CO hydrogenation, and C–C coupling, resulting in production of various (oxygenated) hydrocarbons.^49,52,58,59^

Studies of electrochemical CO_2_RR on Cu surfaces have shown the impact that surface structure has on catalytic activity and product selectivity.^60–64^ Despite the ability of Cu to generate various value-added products via electrochemical CO_2_RR, facilitating the reaction at appreciable current densities on planar Cu surfaces requires large overpotentials. For instance, in one of the early investigations, Hori et al. obtained a total cathodic current density of 5 mA/cm^2^ in an aqueous environment (pH 6.8) on polycrystalline Cu at a potential more negative than −1.4 V vs normal hydrogen electrode (*V*_NHE_), which amounts to approximately 1 V of overpotential.^65^ In more recent and optimized three-electrode cell designs, the current densities of planar Cu electrodes at similar overpotentials increased to around 10 mA/cm^2^, which is far below the requirements for practical implementation.^51^ To enhance the overall CO_2_RR rates on Cu, researchers have focused on nanostructured Cu catalysts. Research efforts in this area include the development of high-surface-area-to-volume-ratio Cu electrocatalysts such as nanoporous films,^66–72^ oxide-derived electrodes,^73–78^ nanoparticles,^79–85^ nanowires,^86–89^ nanoflakes,^90^ roughened surfaces prepared by electrodeposition,^91,92^ or plasma treatments,^93^ as well as Cu-based bimetallic or alloy nanocrystals.^94–98^ Planar and nanostructured Cu surfaces show similar activities when cathodic current densities are normalized by the electrochemically active surface area (based on the estimation of double layer capacitance).^90^ In other words, variations in the roughness factor do not significantly alter the intrinsic activity of Cu. However, CO_2_RR product selectivity has been shown to correlate with the size of the Cu nanoparticles^85^ or the roughness factor of the electrodes.^90^ As an example, for CO reduction in alkaline solutions, increasing the roughness factor of Cu electrodes has been shown to increase selectivity toward multicarbon oxygenates at the expense of the parasitic hydrogen evolution reaction.^90^ However, research on the underlying mechanisms of such enhancement in the selectivity and the interplay between chemical kinetics and transport effects is still ongoing. Enabling the synthesis of repeatable and well-controlled morphologies with various roughness factors (i.e., tailored porosity), which is the focus of this review, can enable studies focused on elucidating the mechanisms responsible for tuned CO_2_RR selectivity.

CO_2_RR technology is still in its infancy when compared with industrialized water electrolysis for hydrogen production.^99^ However, over the past three decades, conversion efficiencies toward several CO_2_RR products, such as CO, ethylene (C_2_H_4_), formate (HCO_2_^–^), and ethanol (C_2_H_5_OH), have increased continuously.^100^ According to a technoeconomic analysis in 2019, CO_2_RR technology could be deemed profitable if electrical-to-chemical conversion efficiencies reach above 60% and renewable electricity costs fall below $0.04/kW h.^100^

The highest efficiencies for the CO_2_RR to date have been achieved in membrane electrode assembly (MEA) electrolyzers, where CO_2_ is delivered as a humidified vapor phase to the cathode. To enable good mass transport of the vapor-phase CO_2_ reactant feed, a gas diffusion electrode (GDE)^101^ consisting of a porous gas diffusion layer substrate upon which a catalyst layer is deposited is used as the cathode. The cathode is separated from the anode via an ion exchange electrolyte membrane that ideally only allows the exchange of certain charged species (ions such as hydronium or hydroxide). CO_2_RR takes place at the cathode, and an oxidation reaction, usually the oxygen evolution reaction (i.e., water oxidation), occurs at the anode. Figure 1a illustrates the schematics of the cathode and anode catalysts separated by a membrane, and Figure 1b shows a nanoporous Cu surface with the corresponding reactants and adsorbed species. Reports of geometric current densities around 1 A/cm^2^ at voltages near 4 V yielding Faradaic efficiencies around and above 50% toward multicarbon products, such as ethylene and ethanol, have been reported for CO_2_RR on nanostructured Cu in MEA configurations.^102–104^

**Figure 1:**
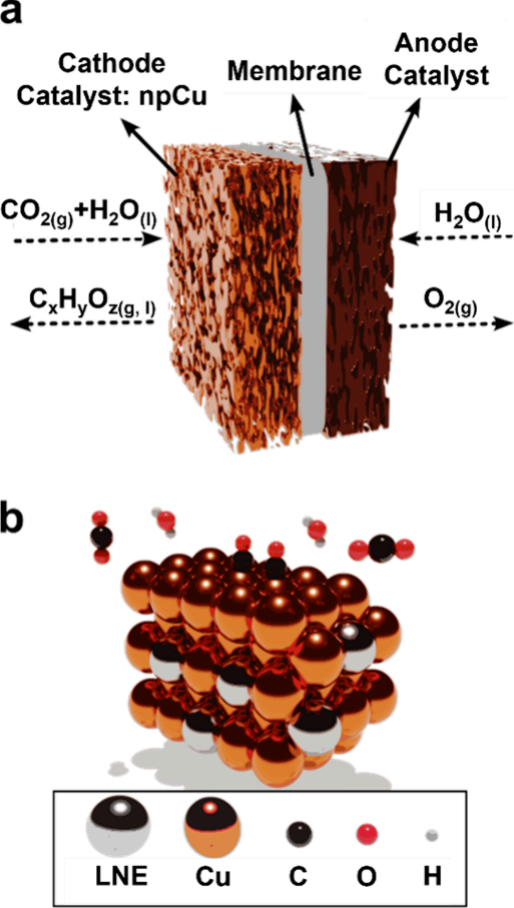
(a) Schematic of a cathode catalyst layer for electrochemical CO_2_ reduction, where mixtures of CO_2_ gas and water vapor are fed into the cathode (nanoporous Cu) to produce various (oxygenated) hydrocarbons after reduction. Oxygen evolution reaction takes place on the anode catalyst (usually IrO_2_), which is separated from the cathode by a membrane. (b) Schematic of the reaction environment on an atomic scale, where CO_2_ and H_2_O molecules are approaching and two *CO are adsorbed on the surface of Cu. The gray atoms represent the remaining less noble element (LNE) after dealloying, which is discussed in section 2.

Porous cathodes are crucial for facilitating mass transport phenomena during the CO_2_RR in MEA. By providing insights into the role of various parameters used in (electro)­chemical techniques to synthesize nanoporous Cu, this review sheds light on the development of well-defined and controllable morphologies with desired porosity to increase the accessibility of electrocatalytic active sites and enhance the selectivity toward value-added products.

### 1.2 Self-Sanitizing Effects of Cu

Microorganisms are rapidly killed on the metallic copper surfaces. In fact, the self-sanitizing attribute of copper has been a known phenomenon since ancient times. A prominent example includes the antifoulant property of Cu on wooden ships, which led to the discovery of cathodic protection in the early 19th century by Humphry Davy and Michael Faraday.^105^ Recently, self-sanitizing surfaces have received renewed attention, especially in the wake of global pandemics such as COVID-19 in the year 2020.^106–108^ Antimicrobial effects of metallic Cu surfaces have been studied in the laboratory sporadically since the early 2000s, as reviewed by Grass et al.^109^ Contact killing has been reported to be even more effective on dry Cu surfaces than on wet surfaces, whereby bacteria inactivation has been reported to take place after a few minutes of contact with a dry Cu surface.^110–112^ The rapid and potent biocidal effects of Cu, particularly those in dry conditions, have further complicated its detailed, molecular, and mechanistic understanding. One of the most interesting aspects of contact killing of microorganisms by Cu is the degradation of genomic and plasmid DNA (see Figure 2). The loss of the genome deprives the organisms of developing resistance due to the lack of the transfer of resistance determinants between generations.^109^ Health care-associated (i.e., hospital-acquired) infections^113–115^ have been effectively reduced by the use of Cu based alloys in the internal design of hospitals and health care facilities. For instance, using components made of Cu in patient rooms reduced infection rates by 58% when compared to rooms with components made of standard materials.^113^

**Figure 2:**
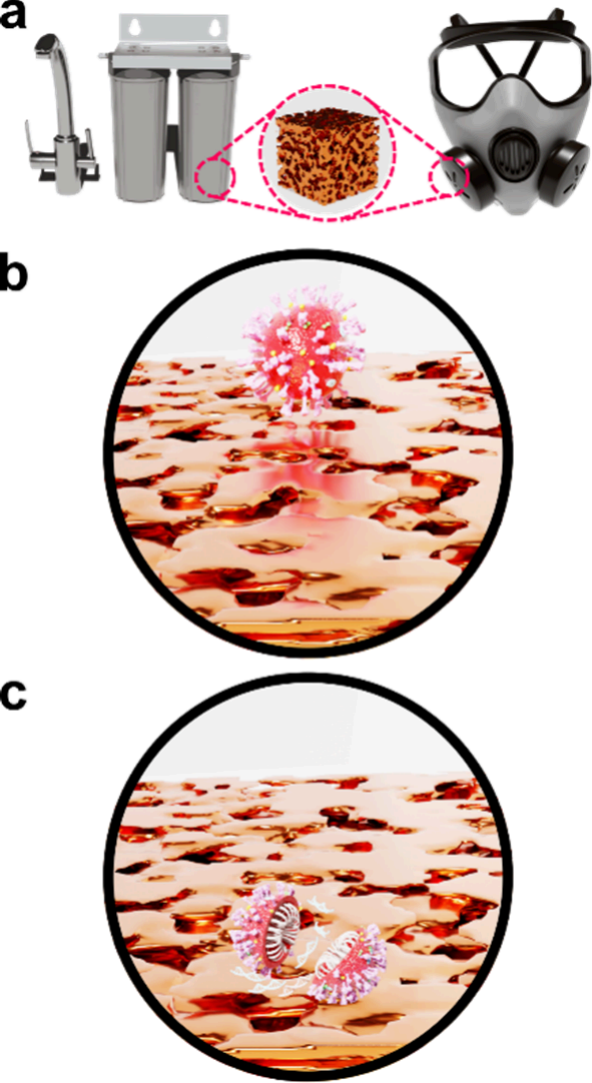
(a) Equipment for water and air filtration with an inset in the middle showing a schematic of nanoporous Cu. (b, c) Schematics of a typical coronavirus on nanoporous Cu before and after contact killing, respectively.

Although the authors could not find many investigations on the biocidal effects of nanostructured Cu to date,^116^ enhancement is expected to arise with increased surface-area-to-volume-ratio, as contact killing is a surface-based process, and thus, this represents an emerging opportunity for research and development. Nanoporous Cu in particular could be envisioned as a filter in disinfecting masks as well as in other disinfection devices. Since different microorganisms and viruses have different sizes (e.g., the coronavirus shows a diameter of around 65 to 125 nm^117^), a deep understanding of the techniques that enable a high level of control over the pore size of the resulting nanoporous Cu is desirable to develop these disinfectant products.

## 2 Synthesizing Nanoporous Cu via Dealloying

Among several methods of fabricating Cu nanostructures, dealloying,^118–120^ i.e., the selective electrolytic dissolution of a less noble element (LNE) from an alloy and the simultaneous redistribution of the more noble element (MNE) on the surface, is one of the most flexible, controllable, and economical methods available to date. The product of dealloying is a metallic nanoporous material consisting of a monolithic ligament-pore structure with nearly zero net curvature; that is, overall, the nanostructure does not exhibit a significant curvature in any direction. The prerequisite for dealloying is that the elements of an alloy must have sufficiently dissimilar metal/metal ion equilibrium potentials, allowing the more active metal species to selectively leach out, while the MNE is redistributed around the surface primarily via surface diffusion or highly local dissolution followed by redeposition (facilitated by partial solvation by water molecules and the anions in the electrolyte) to form an interconnected ligament-pore structure.^118,121^ Figure 3a shows schematics of a surface made of Cu and another less noble (LN) metal, Figure 3b shows the dissolution of the LNE, Figure 3c shows the agglomeration of the Cu atoms after surface redistribution, and Figure 3d shows the resulting nanoporous structure, which is Cu rich on the surface and contains Cu and the remaining LNE in the core of the ligaments. The kinetics of the less-noble element dissolution vs the more-noble element redistribution on the surface determines the size scale of the resulting nanoporous structure after dealloying. Smaller nanoporosity can be obtained if the dissolution of the LNE is expedited or the redistribution of the MNE is hindered. On the other hand, promoting surface redistribution of the MNE, accompanied by slow dissolution of the LNE, results in larger porosity.

**Figure 3:**
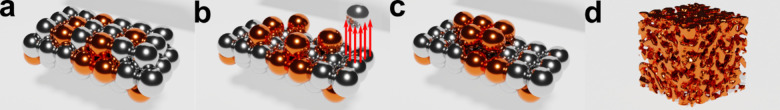
Schematics of (a) a slice of a binary Cu alloy; (b) the dissolution of the less noble element from high energy sites such as kinks and ledges; (c) the initiation of the ligament formation after surface redistribution of the Cu atoms; and (d) an example of the resulting nanoporous structure.

Dealloying has been employed since ancient times by pre-Columbian civilizations, such as the Inca, to fabricate objects with shiny gold surfaces from Au–Cu alloys;^122,123^ however, scientific investigations on dealloying have initially been conducted in the context of materials degradation.^124–127^ The first modern studies on dealloying were performed in the 1860s^128^ on brass and bronze alloys in various aqueous acidic media. Later, nanoporous materials developed from dealloying, in particular Raney metals,^129^ and more recently, nanoporous gold (np-Au)—have found a number of applications in catalysis^130–133^ and sensing,^134–136^ to name a few.

One key parameter for precursor material selection is their standard reduction potential; however, a passivating environment or the presence of various complexants in the dealloying electrolyte can completely change the standard potential. More commentary on this topic can be found in Section 2.4 where the effect of electrolyte on dealloying is discussed. Figure 4 illustrates different metals that are less noble than Cu, excluding radioactive, rare earth, alkaline, and alkaline earth metals (except for Mg), relative to their standard reduction potential and metallic radii. Additionally, the lattice structures at ambient conditions are indicated by distinct marker styles with their edge color mapped to the melting point of each element.

**Figure 4:**
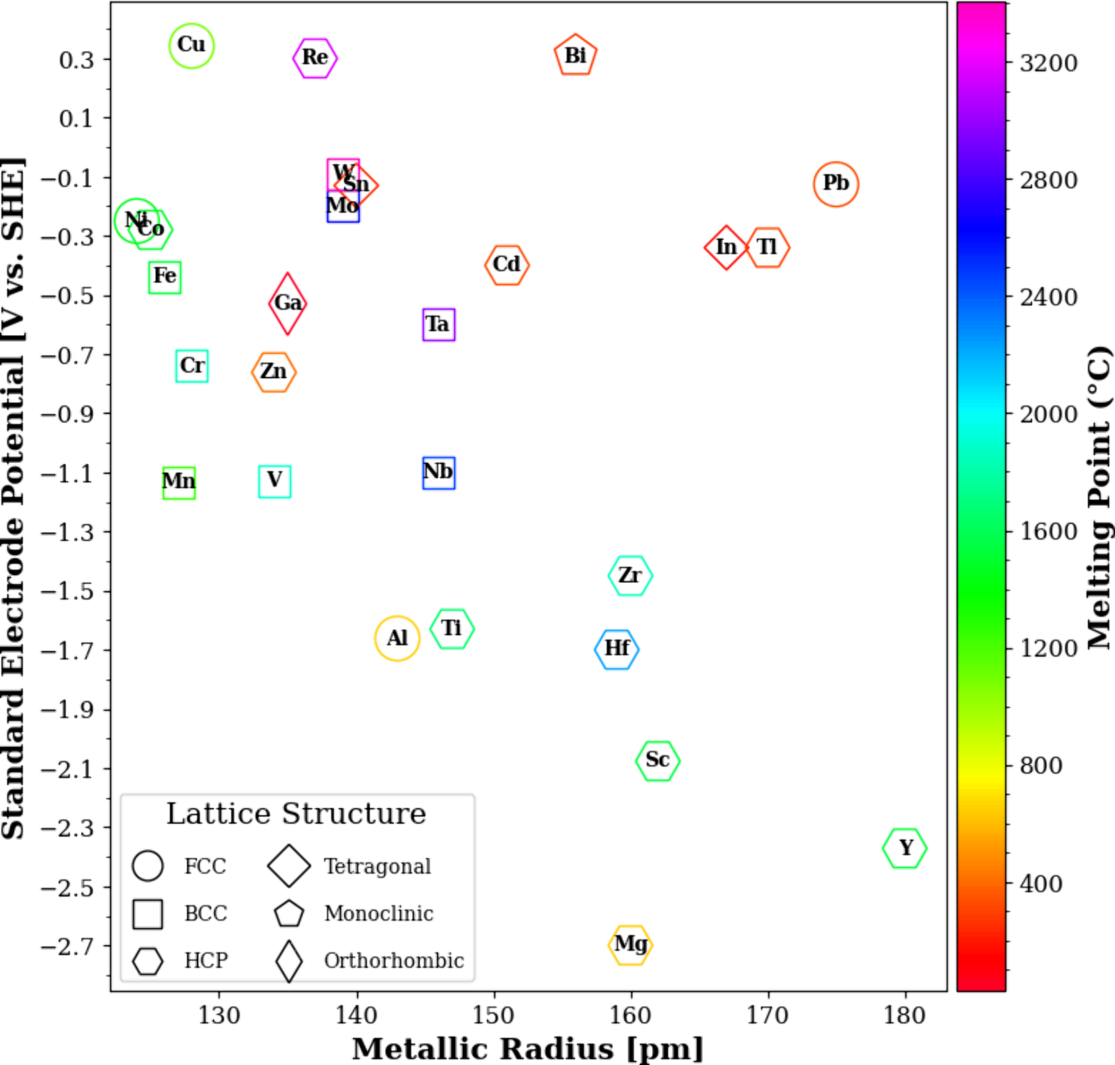
Metallic elements ordered by their radii on the horizontal axis and their standard reduction potential in *V* vs standard hydrogen electrode (SHE) on the vertical axis. The outline color of each marker is mapped to the melting point of the metals. The lattice structures at ambient conditions are indicated by the following marker styles: circles for face-centered cubic (FCC); squares for body-centered cubic (BCC); hexagons for hexagonal closed-pack (HCP); big diamonds for tetragonal; pentagons for monoclinic; small diamond for orthorhombic.

Theoretically, the more similar the metallic radii and the lattice structures of the alloying components, the higher the possibilities of solid solution formation, which is expressed as equilibrium solid solubility and estimated by the Hume–Rothery Rules.^137^ Single-phase precursors, such as solid solutions or intermetallic compounds, are desirable from the perspective of forming a homogeneous and smooth nanoporous structure upon dealloying. The dealloying of intermetallic compounds differs from that of solid solutions by having slower kinetics due to higher energy barriers for surface diffusion, resulting in a smaller porosity length scale.^25,138,139^ Conversely, dealloying multiphase precursors is likely to result in heterogeneous porous morphology with interrupted uniformity and patches of nondealloyed regions. However, such heterogeneity arguably enhances the mechanical stability of the dealloyed materials.

Depending on whether the selective dissolution occurs at open-circuit potential (OCP) or upon the application of an external potential, dealloying methods can be divided into chemical dealloying (using an oxidant, such as nitric acid) and electrochemical dealloying processes, respectively. While the latter provides a higher level of control over the resulting morphology of the dealloyed materials, chemical dealloying is a more facile technique and, consequently, could be more suitable for large-scale applications.

Nanoporous copper (np-Cu) structures with ligament-pore sizes as fine as a few tens of nanometers (mesoporous) and as coarse as several hundreds of nanometers (macroporous) have been developed via chemical dealloying of various Cu alloy precursors, including Mn–Cu,^140–142^ Mn–Al–Cu,^143,144^ Ti–Cu,^145,146^ Zn–Cu,^147–150^ Zr–Cu,^151^ Ni–Cu,^152^ Al–Cu,^133,153–158^ Mg–Cu,^12,159–161^ Al–Mg–Cu,^162^ and Zr–Al–Cu^163^ alloys. The precursors used, their heat treatment history and crystal structure, the chemical dealloying conditions, and the resulting morphologies of the np-Cu structures are summarized in Table 1.

**Table 1:** Summary of np-Cu Structures Developed via Chemical Dealloying in Various Aqueous Electrolytes

precursors								
	fabrication			dealloying				
composition [at %]	method	heat treatment	phase	electrolyte	time	*T* [K]	morphology	ref
Mn_70_Cu_30_	quench furnace technique: held at 1300 °C for 20 min under flowing Ar in an alumina crucible, then furnace cooled to 900 °C in about 20 min	annealed at 900 °C for 72 h and quenched in water at room temperature	Cu, γ-Mn	dilute HCl (pH 1.3)	8 days	RT^a^	a smooth ligament morphology, with an average ligament diameter of 125 ± 30 nm; a residual Mn content of 0.2 at % as determined by EDX	^141^
				1 M citric acid (pH 1.3)	10 days		ligaments with an average diameter of 80 ± 20 nm; not as smooth or uniform as those formed in dilute HCl electrolyte; a residual Mn content of 2.4 at % as determined by EDX	
				1 M (NH_4_)_2_SO_4_ + 0.01 M MnSO_4_ (pH 5)	6 days		ligaments with an average diameter of 53 ± 8 nm; a residual Mn content of 4.4 at % as determined by EDX	
				0.01 M H_2_SO_4_ + 0.001 M MnSO_4_ (pH 1.9)	6 days		very smooth ligaments with an average diameter of 45 ± 11 nm; a residual Mn content of 1.6 at % as determined by EDX	
Mn_85_Cu_15_	voltaic arc heating: melt the charges under an argon atmosphere, and then the melt was cooled down into ingots; the Mn–Cu ingots were remelted by high-frequency induction heating and then melt-spun onto a Cu roller	no additional treatment; used as melt-spun	Cu, γ-Mn	0.075 M H_2_SO_4_	4 h	RT^a^	the least smooth and the least uniform ligaments from among the compositions experimented with; the average ligament diameter is around 200 nm	^140^
Mn_75_Cu_25_							the pore size is about 150 nm with the ligament diameter of 30 ± 20 nm	
Mn_65_Cu_35_							the average pore size is 50 ± 20 nm; but dealloying of Mn_65_Cu_35_ produced bigger ligament diameters than Mn_55_Cu_45_ alloys	
Mn_55_Cu_45_							the average pore size is 50 ± 20 nm	
Mn_65_Cu_35_				0.075 M H_2_SO_4_ with SDBS^c^ as the surfactant	17 h		the average pore size is 10 ± 5 nm	
Mn_55_Cu_45_							fine pores with an average size smaller than 10 nm were obtained	
Mn_70_Cu_30_	melt-spun ribbons with a thickness of ∼20 μm	no additional treatment	Cu, γ-Mn	0.5 M HCl	qualitative: short to long	RT^a^	nanoporosity was formed, however the morphology of the nanoporosity is described as heterogeneous (as opposed to smooth and uniform)	^13^
							longer dealloying times resulted in significant coarsening of the nanostructure; extended immersion times showed that np-Cu samples are unstable in strong acid solutions, as the collapse of the specimens was observed	
				0.1 M HCl	4 h		uniform nanoporosity was achieved after 4 h	
					8 h		the nanoporous structure becomes inhomogeneous as the immersion time is extended to 8 h	
				0.025 M HCl	0.5 h, 2 h, 4 h, 6 h, 12 h, 20 h, 32 h		0.025 M HCl was found to be the optimum concentration to synthesize np-Cu that is stable in the solution for longer times and allows for ligament/pore size tailoring by controlling the dealloying time; a direct and nearly linear relationship is reported between the dealloying time and ligament/pore size (from 0.5 to 32 h, the pore size increased from 15 to 120 nm); the Mn residue in the np-Cu reduced to 9 at % after 0.5 h, 2.7 at % after 2 h, and remained constant at 1.5 at % after 5 h	
				0.01 M HCl			at this low concentration, nanoporosity was still obtained; however, with significantly slower kinetics	
Al_80_Cu_10_Mn_10_	vacuum induction melting technique	no additional treatment	Al_11_Cu_5_Mn_3_AlCu_2_Mn	0.1 M HCl	12 h, 1 day, 2 days, 4 days, 7 days, 10 days	RT^a^	during the dealloying process, a dramatic change of the microstructure has been observed, which could be summarized as the following sequence: corrosion pits → network with ultrafine fibers → aggregated clusters → islands → island-like ligaments → bicontinuous ligaments	^143^
Al_72_Cu_28_	the purchased alloy was recast into rods and quenched	no additional treatment	CuAl_2_ and Al-CuAl_2_ eutectic	6 M NaOH	∼10–12 h	274	a fine network of ligaments with a thin crust on the outside surface; there is extensive cracking on the surface from shrinkage due to loss of material	^153^
Al_78_Cu_22_ film	magnetron sputtered on a Si substrate	no additional treatment	IIM^b^	0.5 M NaOH	5 min	RT^a^	it has clear and uniform ligament/pore bicontinuous channels with an average ligament size of 19 nm	^154^
Al_70_Cu_30_ film							the average ligament size was ca. 25 nm	
Al_63_Cu_37_ film							it seems some of the ligaments were aggregated, while others still preserved nanoscale features; the pore channel of the nanoporous structure was still pronounced, and the average ligament size was ca. 35 nm	
Al_78_Cu_22_ film				0.5 M NaOH	15 s	RT^a^	the thickness of the nanoporous film was around 75 nm; the average ligament size was ca. 18 nm	
					60 s		the average ligament size was ca. 19 nm, which is similar to that of np-Cu films with 15 s of dealloying time; however, the dealloying depth increased to 300 nm	
					5 min		the average ligament size was ca. 25 nm	
Al_70_Cu_30_ film				1 M NaOH, 0.5 M NaOH, 0.01 M NaOH	5 min	RT^a^	the ligament sizes varied from 25 to 32 nm; there was no obvious difference in average ligament size with the use of different concentrations of NaOH; however, a significant amount of Al, up to 20 at % remained in the np-Cu film as the concentration of NaOH decreased to 0.01 M	
Al_60_Cu_40_	by using a single roller melt spinning apparatus, the prealloyed ingots were remelted by high frequency induction heating in a quartz tube and then melt-spunonto a copper roller with a diameter of 0.35 m at a speed of 1000 or 1500 rpm in a controlled argon atmosphere	no additional treatment; used as melt-spun	AlCu	5 wt % HCl	2 h	363 ± 5	a typical porous structure was found; the size of ligaments/channels was between 100 and 300 nm	^155^
Al_67_Cu_33_	charges were melted using a high-frequency induction heating in a quartz crucible, and then the melt was cast into ingots in an iron chill mold; the ingots were remelted again and then melt-spun onto a Cu roller, resulting in 30–60 μm thick ribbons; in addition, the ingots were also cast into rods and slices by blow casting to be used in the two-step dealloying	no additional treatment	Al_2_Cu; Al_2_Cu and AlCu	5 wt % HCl	2–4 h	363 ± 5	the porous structures contain nanoparticles with sizes ranging from one to several hundred nanometers; moreover, large particles with a size up to 1 μm were also observed	^156^
Al_65_Cu_35_							the ribbons consist of nanoparticles and some ligaments with sizes between 100 to 300 nm; no microcracks were observed on the top surface	
Al_60_Cu_40_							an open, three-dimensional bicontinuous interpenetrating ligament-channel structure with length scales of 100–300 nm was observed	
Al_50_Cu_50_							a uniform porous structure with length scales between 300 and 500 nm was observed	
Al_65_Cu_35_				two-steps dealloying: (I) 20 wt % NaOHat RT^a^ and then at 363 ± 5 K (II) 5 wt % HCl at 363 ± 5 K	1–2 h		many cracks (10 s of μm in length and sub-μm in width) can be observed on the surface of the ribbons; moreover, the margin of the ribbons shows a typical ligament-channel structure, differing from that of the center of the ribbons	
Al_60_Cu_40_							a uniform ligament-channel structure can be obtained; the uniform porous structure runs throughout the whole ribbons	
Al_50_Cu_50_							large length scales of ligaments/channels vary from 100 to 500 nm	
Al_75_Cu_25_	melting pure copper and aluminum in quartz crucibles by voltaic arc heating in an Ar atmosphere, then slowly cooled down (equilibrium solidification); the wire was cut into 200 μm slices and polished	no additional treatment	Al and CuAl_2_	1 M NaOH	until no more bubble was seen	RT^a^	a periodic structure of alternating channels and walls in the scale of hundreds of nm among elliptical island-like structures in the scale of several μm, both of which are comprised of an open, 3D nanoporous structure with pores and ligaments of 10–50 nm wide	^158^
Al_67_Cu_33_ with NaCl (20 wt % of the Al–Cu powder mixture)	powder metallurgy method: (I) raw Al and Cu powders were first mixed and ground; (II) milled NaCl with a particle size of ∼70 μm was added to mixed Al–Cu powders; (III) the well-mixed powders were cold-pressed; (IV) the green compacts were continuously sintered in a pipe at 500 °C for 30 min, and then the as-sintered bulk alloys were desalinated at 90 °C for 3 h in a water bath to dissolve NaCl	no additional treatment	CuAl_2_ and AlCu	10 wt% NaOH	10–50 h	RT^a^	it has an open, bicontinuous, spongy-like morphology with the ligament sizes of approximately 40–80 nm and the pore sizes of about 30–50 nm	^128^
Al_67_Cu_33_ with NaCl (20 wt % of the Al–Cu powder mixture)				5 wt% HCl			the hierarchical structure combines porosity on distinctly different size scales of 40 nm and 1 μm	
Al_67_Cu_33_ without NaCl				5 wt % HCl or 10 wt % NaOH			nanostructured particles with various sizes from 60 to 80 nm	
Al_80_Cu_20‑x_V_*x*_ (x = 0, 0.2, 1, 2)	melt-spun ribbons with ∼50 μm thickness and ∼5 mm width made by arc melting from pure ingots in a vacuum	no additional treatment	α-Al and CuAl_2_	Ar-purged 2 M NaOH aqueous electrolyte	2 h	IIM^b^	uniform bicontinuous nanoporous architecture consisting of interconnective metallic ligaments and bimodal nanopore channels with characteristic lengths of ∼120 nm first-order pores and ∼15 nm secondary pores	^68^
θ-phase Al_2_Cu ingot	melting pure metal powders in a graphite crucible in an induction furnace by holding them at 750 °C for 10 min before cooling down naturally in the furnace; the resulting ingot was then crushed into 2 mm particles and then ball-milled with 2 mm zirconia grinding balls at 2000 rpm for 8 h until a fine powder with a particle size of less than 10 μm was obtained	no additional treatment	θ-phase Al_2_Cu	6 M KOH	until no more bubble was seen	IIM^b^	bicontinuous nanoscale ligament-pore morphology with ∼30 nm ligaments and pores	^70^
Zn_70_ Cu_30_	heating Cu powders and bulk Zn with an atomic ratio of 3:7 in a nitrogen atmosphere	raised to 900 °C and held for 2 h, then cooled down to 530 °C to anneal for 2 h, finally cooled down to ambient temperature	Cu_5_Zn_8_ and CuZn_5_	1 M HCl + 5 M NH_4_Cl	IIM^b^	343	the channel size and ligament width of np-Cu were 210 ± 30 and 120 ± 30 nm, respectively	^148^
				two steps: (I) dealloying in 1 M HCl + 5 M NH_4_Cl; (II) electroless plating in 10 g/L CuSO_4_·5H_2_O + 40 g/L EDTA^d^ + 15 mL/L (CH_2_O + NaOH); pH 11	24 h		after dealloying followed by electroless plating, a three-dimensional bicontinuous porous structure with channel and ligament sizes of 150 ± 30 nm was developed; additionally, the mechanical properties of the np-Cu improved after the electroless plating step	
Zn–Cu surface alloy	electrochemical deposition of Zn was done at 20 mA/cm^2^ in 60 g/L ZnCl_2_, 200 g/L KCl, 25 g/L H_3_BO_3_, and 20 mL/L LuZn-8 additive	treated at 100 °C for 2 h in N_2_ gas	CuZn_5_ and CuZn	5 wt % NaOH	4, 12, and 24 h	RT^a^	after 4 h of dealloying, the surface showed a discontinuous structure composed of 30 nm particles	^164^
							after 12 h, a rough structure with several small islands was obtained	
							after 24 h, three-dimensional porosity was obtained with uniform pores of about 10 nm and ligament size of ∼15 nm	
Zn–Cu surface alloy	electrochemical deposition at 20 mA/cm^2^ for 30, 60, and 120 s in a solution of 30 g/L ZnCl_2_, 150 g/L KCl, and 5 g/L cetyltrimethylammonium bromide (CTAB) on a commercial Cu foil that was electropolished in 85 wt % phosphoric acid at 0.5 V_Ag/AgCl_ for 500 s	treated at 150 °C for 4 h under an Ar atmosphere	Cu–Zn alloy species	1 M KOH	6, 12, and 24 h	RT^a^	only the sample that was electrodeposited for 120 s showed a 3D porous structure	^66^
Zn–Cu surface alloy	electrochemical deposition at 10 mA/cm^2^ for 20 min	treated under an Ar atmosphere	pure cubic phase of Cu_5_Zn_8_	pH 4, HCl	24 h	298	a three-dimensional interconnected porous nanostructure with a pore size of about 100 nm	^67^
Zn–Cu thin films	electrochemical deposition on Au/Cr/Si substrate by chronoamperometry	no additional treatment	IIM^b^	5 wt % NaOH for 15 h, then 11.3 M HCl for 30 s		IIM^b^	μm-sized holes were formed on the surface of the films.	^147^
				2 wt % NaOH for 15 h, then 11.3 M HCl for 20 s			the number of μm-sized holes was dramatically reduced. However, the pores were still larger (>500 nm) than expected.	
				2 wt % NaOH for 15 h, then 4 M HCl for 20 s			on reducing the concentration of HCl to 4 and 3 M, the size of the ligaments and pores reduced to 100 nm, with a more uniform pore-size distribution	
				2 wt % NaOH for 15 h, then 3 M HCl for 20 s				
				2 wt % NaOH for 15 h, then 3 M HCl for 60 s			the porous structures were distributed over the entire thickness of the film	
Ti_30_Cu_70_	arc melting was used to prepare binary Ti–Cu alloys under an Ar atmosphere; the melt spinning method was used to prepare ribbons	no additional treatment; used as melt-spun	Ti_2_Cu_3_	0.027 M HF (pH 3.3)	43.2 ks	298	nanoporosity was not obtained except for a very thin film on the surface	^146^
				0.133 M HF (pH 2.6)			nanoporosity was obtained	
Ti_40_Cu_60_, Ti_50_Cu_50_, Ti_60_Cu_40_			amorphous	0.027 M HF (pH 3.3)0.133 M HF (pH 2.6)			dealloying in a higher concentration of HF solution resulted in a coarser nanoporous structure	
Ti_50_Cu_50_				0.027 M HF (pH 3.3)	1 h	298	the np-Cu structures formed in three solutions are similar in morphology, and the length scale of the ligaments is larger in more concentrated HF solutions	
				0.133 M HF (pH 2.9)				
				0.651 M HF (pH 2.6)				
				0.133 M HF (pH 2.9)	10 min, 0.5 h, 1 h, 3 h		with increasing immersion time, the length scales of the bicontinuous, i.e., the ligaments and the pore sizesincreased from tens of nanometers to hundreds of nanometers	
				1 M HCl + 1 M HNO_3_	15 days		nanoporosity was not obtained. Only pitting was observed on the surface; EDX analysis showed the presence of Ti_2_O on the surface	
Ti_60_Cu_40_, Ti_50_Cu_50_	arc melting was used to prepare binary Ti–Cu alloys under an Ar atmosphere	no additional treatment; used as melt-spun	amorphous	0.03 M HF0.13 M HF	3 h	298	the mean values of nanopores of Ti_60_Cu_40_ and Ti_50_Cu_50_ ribbon alloys were 37 and 31 nm in 0.03 M HF solution, and 86 and 185 nm in 0.13 M HF solution, respectively; the mean values of ligament sizes were found to be 46 and 57 nm in 0.03 M HF solution, and 94 and 185 nm in 0.13 M HF solution, respectively	^145^
Ti_60_Cu_40_				0.13 M HF		323	coarser nanoporous structures were obtained at higher temperatures; the residual Ti contents in the nanostructures were also slightly higher after dealloying at higher temperatures	
Ti_40_Cu_60_						348		
Mg_67_Cu_33_	the charges were melted in a quartz crucible, and the Mg–Cu melts were cast into an iron-chill mold; remelted by high-frequency induction heating and melt-spun onto a Cu roller, resulting in ribbons with a thickness of 30–70 mm	no additional treatment; used as melt-spun	Mg_2_Cu	5 wt % HCl	0.5 h	First, at RT^a^ and then at 363 ± 5	the mean length scale of the ligament-channel structure was 148 ± 35	^159^
Mg_60_Cu_40_							the mean length scale of the ligament-channel structure was 175 ± 27	
Mg_50_Cu_50_			Mg_2_Cu and MgCu_2_				the mean length scale of the ligament-channel structure was 211 ± 37	
Mg_40_Cu_60_							the mean length scale of the ligament-channel structure was 272 ± 63	
Mg_33_Cu_67_			MgCu_2_				dealloying was incomplete and only was seen at the margins, indicating that the parting limit lies between 60 and 67 at % Cu	
Mg_80.7_Cu_19.3_	an alloy of Mg and Cu was deposited on Cu foil via ion-beam sputtering	no additional treatment	IIM^b^	10 mM NH_4_Cl	5, 10, and 15 min	303	a typical nanoporous structure was obtained; larger pores and ligaments were formed as the dealloying time increased	^161^
Mg_70_Cu_20_Y_10_, Mg_65_Cu_25_Y_10_, Mg_60_Cu_30_Y_10_, Mg_50_Cu_40_Y_10_	Cu–Y prealloys were produced by arc-melting under a Ti-gettered Ar atmosphere; then the prealloys were remelted with Mg pieces in a high-frequency induction furnace under a high-purity Ar atmosphere; then, glassy ribbons with thicknesses of 30–60 mm were melt-spun on a rotating Cu roller	no additional treatment; used as melt-spun	amorphous	0.04 M H_2_SO_4_	90 min	298	the np-Cu exhibited homogeneous three-dimensional (3D) nanoporous morphology with continuous pore channels and solid ligaments; the nanoporous structure coarsened as the Cu content in the precursor alloy increased; the ligament sizes ranged between 30 and 100 nm; the mean ligament size and the Cu content of the precursors have a direct linear relationship; conversely, the volume fraction of the nanopores and the Cu content of the precursors have an indirect linear relationship	^160^
Mg_65_Cu_25_Y_10_					10 min, 30 min, 90 min, 50 h	298	the ligaments coarsened quickly with the prolongation of the leaching time; after 90 min, the mean ligament size of the np-Cu reached 80 nm; moreover, when the leaching time increased up to 300 min, the nanoporous structure started collapsing, and some ligaments grew up into relatively big nanoparticles with sizes close to 200 nm	
Mg_65_Cu_25_Y_10_					20 min	298	all of the np-Cu obtained show uniform continuous nanoporosity; the ligament size exhibits a significant dependence on leaching temperature, increasing from ∼48 to ∼158 nm with increasing temperature from 298 to 363 K	
						323		
						343		
						363		
Zr_47_Cu_48_Al_5_	arc melting in an Ar atmosphere	dried at 50 °C for 1 h	amorphous	0.5 M HF	24 h	RT^a^	NP-Cu structures with pores ranging from 50 to 500 nm decorated with nanocubes of Cu_2_O	^163^

a Room temperature.

b Information is missing.

c Sodium dodecyl benzene sulfonate.

d Ethylenediaminetetraacetic acid.

Electrochemical dealloying has been also extensively applied to fabricate np-Cu from Cu alloy precursors, including Mn–Cu,^13,140,141,165,166^ Mn–Cu–Si,^167^ Mn–Al–Cu,^168^ Zn–Cu,^169–171^ Zr–Cu,^151^ Mg–Cu,^172^ and Mg–Al–Cu^162^ alloys. The precursors used, the electrochemical dealloying conditions, and the resulting morphologies of the np-Cu structures in previous studies are summarized in Table 2. It is noteworthy that the smallest porosity size for np-Cu among these reports is greater than 10 nm.^141^ However, porosities around 5 nm have been achieved in other noncopper systems that have been extensively studied, such as np-Au.^173^ Therefore, a lower limit of ligament-pore size might also be attainable in np-Cu, but not as yet.

**Table 2:** Summary of np-Cu Structures Developed via Electrochemical Dealloying in Various Aqueous Electrolytes

precursors				dealloying					
	fabrication								
composition [at %]	method	heat treatment	phase	electrolyte	applied potential	time	*T* [K]	morphology	ref
Mn_70_Cu_30_	quench furnace technique: held at 1300 °C for 20 min under flowing Ar in an alumina crucible, then furnace cooled to 900 °C for about 20 min	annealed at 900 °C for 72 h and quenched in water at room temperature	Cu, γ-Mn	0.01 M H_2_SO_4_ + 0.001 M	–0.11 V_MSE_	14 h	RT^a^	the ligament structure is the same as obtained from free corrosion, but the dimensions are smaller by a factor of 3, with an average ligament diameter of 16 ± 4 nm	^141^
				MnSO_4_ (pH 1.9)					
Mn_65_Cu_35_, Mn_55_Cu_45_	voltaic arc heating: melt the charges under an Ar atmosphere, and then the melt was cooled down into ingots	no additional treatment; used as melt-spun	Cu, γ-Mn	0.075 M H_2_SO_4_	–0.2 V_SCE_	0.5 h	RT^a^	the pore sizes increased as the Cu content in the precursor alloys decreased	^140^
Mn_55_Cu_45_	the Mn–Cu ingots were remelted by high-frequency induction heating and then melt-spun onto a Cu roller				0 V_SCE_			the average pore size is ∼500 nm	
					–0.2 V_SCE_			the finer and smaller pores were produced at −0.2 V_SCE_ than 0 V_SCE_ with the pore size of several nanometers	
Mn_25_Cu_75_	chill cast; hot-rolled at 800 °C to a thickness of 0.63 cm	annealed at 700 °C for 1 h, milled, cold-rolled to 0.10 cm, annealed at 725 °C for 1 h, and water quenched	Cu, γ-Mn	0.5 M NaCl	ranging from −0.25 to +0.50 V_SHE_	20 h	298 ± 0.05	at the corrosion potential of around −0.05 V_SHE_ for the Mn_25_Cu_75_ alloy, the solution contains around 40% Mn, and the specimen exhibits moderate dealloying; as the potential is shifted to around −0.25 V_SHE_, the manganese content of the solution rises to around 95%, and Cu dissolution is almost completely prevented.	^165^
Mn_50_Cu_50_	chill cast; homogenized at 750 °C for 16 h, hot-rolled at that temperature to a thickness of 0.63 cm	annealed at 750 °C for 2 h, water quenched, and milled; the alloy was then cold-rolled to 0.10 cm thickness; the alloy was then again annealed at 750 °C for 2 h, followed by water quenching to yield the single phase (γ) condition; the two-phase (γ + α -Mn) condition was produced by further annealing selected specimens for 4 h at 450 °C	Cu, γ-Mn or Cu, γ + α-Mn		ranging from −0.60 to +0.25 V_SHE_			the Mn_50_Cu_50_ alloy shows different behavior and much more extreme dealloying; from −0.60 to −0.25 V_SHE_, only Mn is found in solution.	
								the dealloyed surface of the Mn_50_ Cu_50_ alloy was much more porous than that of the Mn_25_Cu_75_ polarized at +0.25 V_SHE_.	
Mn_60_Cu_40_	melting and rapidly quenching process	a long annealing^c^ followed by rapid quenching	Cu, γ-Mn	0.1 M H_2_SO_4_ + 0.001 M MnSO_4_ (treated with NH_4_SO_4_ solution after dealloying)	–0.5 V_MSE_ ^d^	IIM^b^	RT^a^	well-ordered bicontinuous np-Cu with uniform porosity of around 20 nm was obtained; a slight amount of Mn residue was detected by EDS	^166^
Mn_70_Cu_30_	melt spinning method to prepare the precursor ribbons	no additional treatment; used as melt-spun	Cu, γ-Mn	0.001 M HCl	0 V_Ag/AgCl_ (∼0.2 V_SHE_)	IIM^b^	RT^a^	instead of the formation of np-Cu, nanostructured cuprous oxide (Cu_2_O) is formed	^13^
					0.1 V_Ag/AgCl_			when the etching potential was increased to 0.1 V_Ag/AgCl_, the Cu_2_O nanocubes changed to prolate spheroid nanoparticles of Cu_2_O; nanoporosity did not evolve	
Mn_70_Cu_30_, Mn_70_Cu_29.5_Si_0.5_, Mn_70_Cu_29_Si_1_, Mn_70_Cu_28_Si_2_	alloy ingots were prepared by induction melting in Al_2_O_3_ crucibles under a vacuum level of about 5 × 10^–4^ Pa; ribbon samples were made by means of single-roller melt-spinning in a vacuum	no additional treatment; used as melt-spun	Cu, γ-Mn	0.05 M HCl (nondeaerated, i.e., open to air)	ranging from −0.5 to −0.7 V_SCE_	IIM^b^	298	electrochemical dealloying at −0.55 V_SCE_ resulted in an np-Cu wide ribbon with a pore size of ∼30–50 nm	^167^
								the grain size of the melt-spun ribbons was not uniform and varied in the range of a few μm to nm sized grains; wider intergranular mud cracks were found in the case of μm length scale grain boundaries	
Mn_70_Cu_25_Si_5_			Cu, γ-Mn, and precipitation of ß(Mn, Si) primitive cubic					a glue-like structure was formed continuously along the grain boundaries in Si-containing samples, the mechanical integrity of which was improved; the EDX spectrum of this glue-like structure revealed a Mn and Si content of ∼8 and 3.5 at %, respectively; the average Si content in the nanoligaments was less than 1 at %	
Mn_50.2–42.8_Cu_44.9–54.2_Al_4.9–3.0_	hot rolled followed by cold rolling	no additional treatment; used as cold-rolled	Cu, γ-Mn	0.3 M NaCl	–0.2 V_SCE_	2 h	RT^a^	the pore size is about 1 μm	^168^
		annealed at 850 °C for 2 h						the pore size is about 0.1 μm, smaller than that of the dealloyed cold-rolled specimen, because annealing reduced the phase inhomogeneity	
Zr_30_Cu_70_ thin films	magnetic sputtering: deposited on microscope glass slides from a cast Cu_70_Zr_30_ target	no additional treatment	Cu_8_Zr_3_	0.1 M HCl	–0.2 V_SCE_	10 min	RT^a^	the distribution of the elements Cu and Zr is significantly homogeneous in nanocrystalline dual-phase Cu_70_Zr_30_ films; porous Cu film with a 500 nm pore size can be obtained	^151^
			Cu_10_Zr_7_			1 h			
Zn–Cu surface alloys	galvanostatic electrodeposition of Zn on Cu foils in a deep eutectic solvent made from choline chloride and urea with a 1:2 molar proportion, respectively, containing 0.1 M ZnO at different deposition temperatures, current densities, and charge density	no additional treatment	CuZn_5_	0.1 M ZnO + choline chloride-urea (1:2 molar ratio) deep eutectic solvent	–0.40 V_Ag/AgCl_	15 s	353	Cu–Zn surface alloys prepared at a lower deposition temperature with higher current density and charge density, accompanied by a higher dealloying temperature, facilitate the fabrication of a well-organized nanoporous structure	^169^
						45 s	373		
			Cu_4_Zn			300 s	393		
						1000 s			
Zn–Cu surface alloys	galvanostatic electrodeposition of Zn on Cu substrates in ZnCl_2_ and 1-ethyl-3-methylimidazolium chloride (1:1 molar ratio) solution	no additional treatment	Cu_5_Zn_8_CuZn_2_	ZnCl_2_ + 1-ethyl-3-methylimidazolium chloride (1:1 molar ratio)	ranging from +0.1 to 0.5 V_Zn/Zn_^2+^	300 s	393	smooth nanoporous structure with 100 nm-thick ligaments was obtained at an anodic overpotential of 0.35 V	^174^
Cu_*x*_Zn_(100–*x*)_ where *x* = 12–42 and Cu_31_Zn_66_Ni_3_	electrodeposition on Au at −2 V_MSE_ in 100 mM K_4_P_2_O_7_, 7 mM NaH_2_PO_4_ and CuSO_4_ and ZnSO_4_ (and NiSO_4_) for charge densities ranging from 1 to 3 C/cm^2^	no additional treatment	IIM^b^	100 mM NaClO_4_ + 1 mM HClO_4_	sweeping the potential from −1.7 to −0.25 V_MSE_ at 1 mV/s	IIM^b^	RT^a^	larger ligaments were obtained in the presence of Cl^–^ (73 ± 24 nm), followed by SO_4_^2–^ (32 ± 6 nm), and ClO_4_^–^ (21 ± 4 nm).	^171^
				100 mM Na_2_SO_4_ + 1 mM H_2_SO_4_				increasing the amount of Cu in the precursors yielded larger ligaments and fewer mudcracks.	
				100 mM NaCl + 1 mM HCl				the presence of 3 at % Ni in the precursor yielded substantially refined ligaments (12 ± 2 nm).	
Mg_67_Cu_33_	the charges were melted in a quartz crucible with protective fluxes using a resistance furnace; the Mg–Cu melts were cast into an iron-chill mold, and rod-like ingots of 10 mm in diameter were obtained; then, a melt-spinning apparatus was used to prepare rapidly solidified Mg–Cu alloy ribbons	no additional treatment; used as melt-spun	Mg_2_Cu	0.2 M NaCl	–0.3 V_Ag/AgCl_	IIM^b^	RT^a^	the microstructure shows a porous structure; the ligament-channel structure is not obvious, and many Cu nanoparticles with sizes of 250 ± 30 nm can be observed	^172^
Mg_60_Cu_40_			Mg_2_Cu					the microstructure is similar to that of the as-dealloyed Mg_67_Cu_33_ alloy, but the sizes of Cu nanoparticles are 180 ± 30 nm	
Mg_50_Cu_50_			Mg_2_Cu and MgCu_2_					the microstructures show a typical bicontinuous interpenetrating ligament-channel structure with sizes of 188 ± 45 nm	
Mg_40_Cu_60_			MgCu_2_ and a little Mg_2_Cu					the microstructures show a typical bicontinuous interpenetrating ligament-channel structure with sizes of 200 ± 60 nm	
Mg_33_Cu_67_			MgCu_2_					the microstructure exhibits a typical nanoporous structure; the ligaments are flakelike, and the sizes of the ligaments are much smaller than those of the channels (120 ± 30 nm)	
Al_70_Cu_18_Mg_12_(Al_75_Cu_17_Mg_8_)_97_Ni_3_	melt spinning technique to make ribbons	no additional treatment; used as melt-spun	amorphous	1 M HCl	–0.4 V_SCE_	30 s	298 K	a crystalline, Cu-rich, nanoporous structure with a pore diameter of 10–30 nm; the nanoporous structure is finer (e.g., 10 nm) in the alloy containing Ni	^162^
						200 s			
						1000 s			

a Room temperature.

b Information is missing.

c The information about the annealing temperature, duration of annealing, and the temperature of quenching is missing.

d Mercury sulfate electrode.

The ligament-pore morphology and length scale of (electro)­chemically induced metallic nanoporous materials can be manipulated by tuning several parameters, including precursor composition and heat treatment, as well as dealloying potential, electrolyte composition, temperature, and duration. The following discussion attempts to characterize the influential factors and solicit insight from the literature for further facilitation and enhancement of the development of np-Cu from the dealloying of Cu alloys.

### 2.1 Effect of Precursor Composition

The compositional change influences the balance between the dissolution kinetics of the LNE and the surface diffusion kinetics of the MNE. This influence can be measured by the electrochemical polarization of the precursors and observing the variations of the critical potential^175^ at which nanoporosity development starts, which is characterized by a sudden increase in the current density^176^ (see Figure 5). In general, the higher the percentage of LNE in the starting alloy, the lower the potentials required for nanoporosity development. Accordingly, among Mn–Cu alloys, for example, at similar applied potentials, the ones with higher Mn content show faster dealloying kinetics.^165^

**Figure 5:**
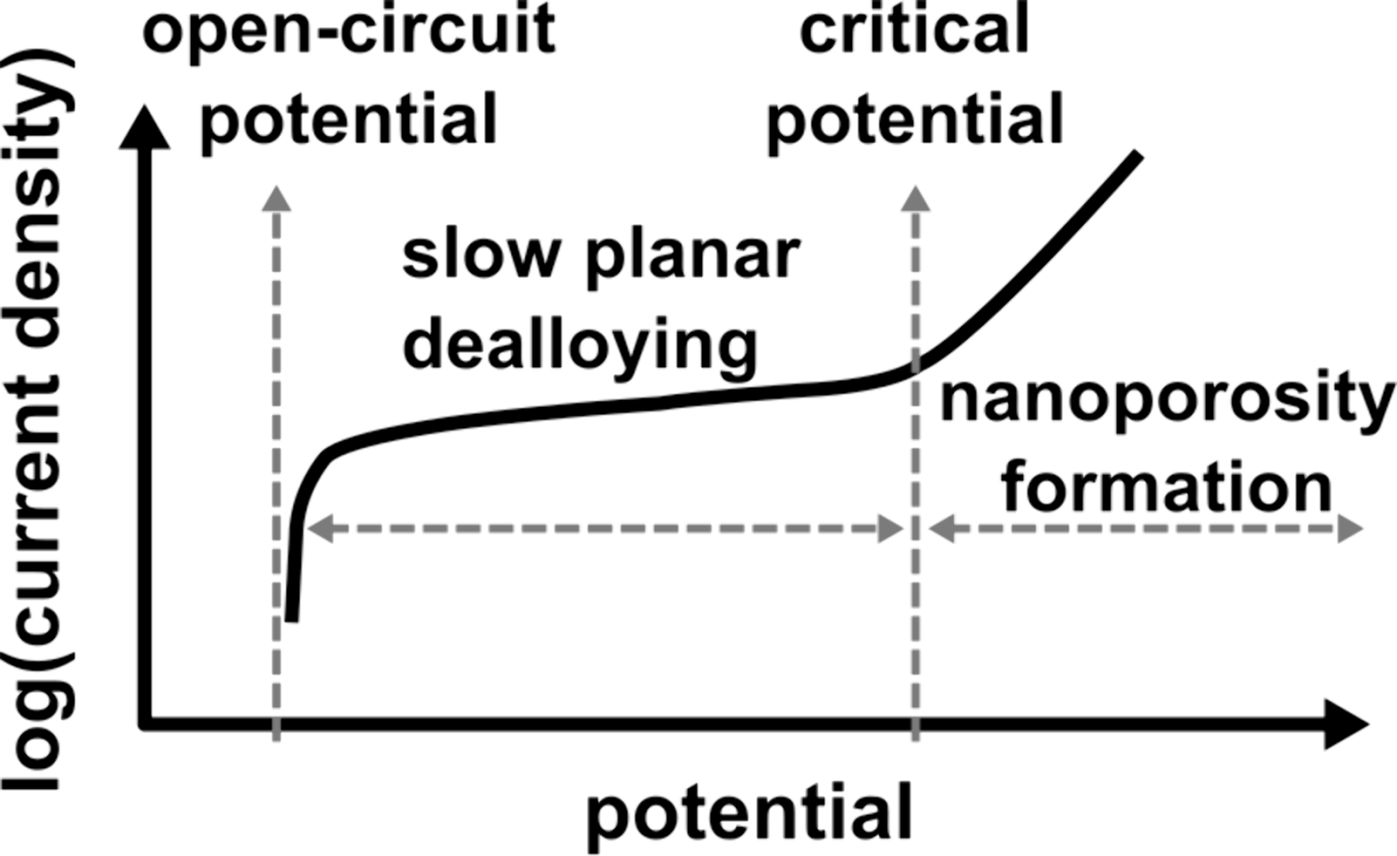
Schematic of a typical anodic polarization curve on a binary alloy composed of constituents with sufficient difference in their standard reduction potential. The vertical axis represents the logarithm of the current density, and the horizontal axis represents the potential. The curve highlights a slow planar dealloying region from open-circuit potential to the critical potential, and the region beyond the critical potential, where dealloying leads to nanoporosity formation.

The kinetics of dealloying for binary Cu alloys is directly impacted by the difference between the standard reduction potential of the alloying elements; the higher the difference, the faster the kinetics of dealloying, assuming all the other influential parameters, such as the electrolyte and the passive/active behavior of the LNE, anion adsorption, applied potential, and temperature, are the same. For instance, the most rapid dealloying is observed in the case of Mn–Cu alloys, followed by Zn–Cu and Ni–Cu alloys, as the difference in the standard reduction potential decreases in this order.

There is a lower limit for the concentration of the LNE below which dealloying cannot proceed due to the lack of a percolating path, i.e., the lack of a continuous connection among the atoms of the LNE throughout the sample, which results in incomplete dealloying (Figure 6a). The site percolation threshold for a face-centered cubic (FCC) binary alloy, for instance, is around 20 atomic (at) % of the LNE.^177–180^ In other words, if a binary FCC alloy has fewer than 20 at % of the LNE, then its complete dealloying would be impossible if the atoms of the MNE were stationary. For example, dealloying studies on a range of compositions of Mg–Cu alloys showed that for Mg_33_Cu_67_ precursor (FCC, solid solution), i.e., when the Mg concentration is near the percolation threshold, the dealloying can only be seen at the sample’s margins, indicating that the selective dissolution can only reach a certain depth and cannot proceed fully.^159,181^

**Figure 6:**
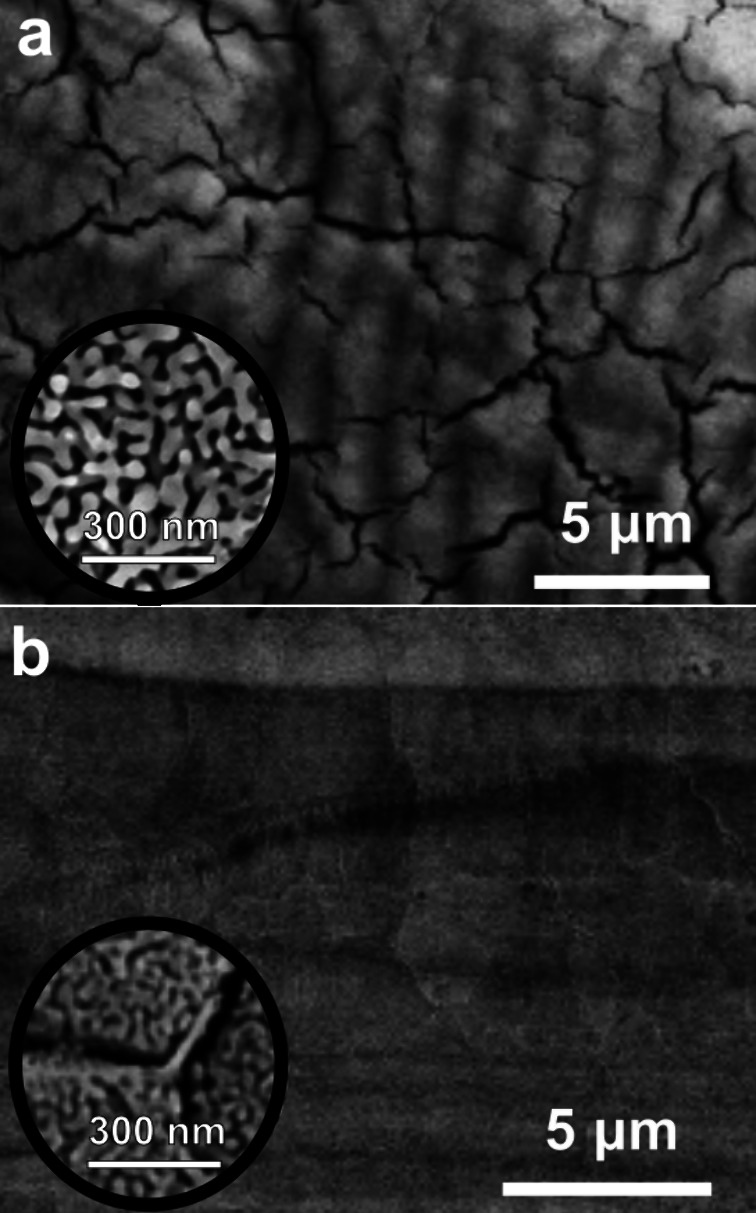
Scanning electron microscopy (SEM) images showing (a) high density of mudcracks in the nanoporous Cu ribbon made from Mn_70_Cu_30_; the bottom-left inset SEM image shows a higher magnification of this dealloyed sample indicating the bicontinuous nanoporous structure with a pore size of ∼30–50 nm; (b) the suppressed mudcracks in the np-Cu sample made from γ-Mn_70_Cu_28_Si_2_; the bottom-left inset SEM image shows a higher magnification of this dealloyed sample indicating the grain boundaries where the Si-enriched “glue” structure is visible. Figure reproduced with permission from Elsevier, copyright 2023.^167^

However, the empirical values for this lower limit, also known as the dealloying threshold or parting limit, often deviate from the theoretical calculations of the percolation threshold.^182,183^ For instance, in the case of the well-studied binary silver–gold alloys (FCC, solid solution), experiments have shown that the dealloying threshold is in the range of 55–60 at % Ag, as opposed to the 20 atom % predicted by the percolation theory. Dealloying of precursors with compositions above the percolation threshold but slightly below the dealloying threshold often results in the sole dissolution of the grain boundaries and fails to yield a nanoporous morphology.^140,184^ Such deviations between the percolation and the dealloying thresholds are mainly attributed to the practicalities around the dissolution of the LNE, namely the fact that the solvation of the LNEs with coordination numbers greater than nine is impractical, hence their dissolution is prevented^182^ (see Figure 6b). In fact, once one-atom-wide percolating paths are excluded from threshold calculations, the resulting theoretical value further approaches the empirical values. Additionally, since in the percolation calculations the atoms are assumed to be distributed randomly and to be immobile, short-range ordering, clustering,^185,186^ and the surface diffusion of the MNE also contribute to varying the lower limit of the LNE needed in practice compared to the percolation prediction. Considerations of the surface diffusion have resulted in a better agreement between the percolation and the dealloying thresholds.^182^

It is worth mentioning that dealloying at much higher temperatures (homologous temperatures around 0.5) in molten salts has shown deviation from the above-mentioned behaviors. For example, by dealloying Fe–Ni systems at 700 °C in molten chloride salts, Ghaznavi et al.^26^ found that the parting limit is geometric in nature and is consistent with percolation predictions of 22% at such conditions. Additionally, lattice diffusion, as opposed to surface diffusion, has been proposed to play a dominant role in the dealloying mechanism of such systems at 700 °C.

Once the precursor composition is within a range viable for dealloying and nanoporosity evolution, it can be manipulated to influence the size of the ligament-pore structure in the resulting nanoporous material. Characteristic studies on dealloyed Mn–Cu,^140^ Mg–Cu,^159,160^ Ti–Cu,^146^ and Al–Cu^156^ precursors revealed a general pattern, according to which the porosity length scale of the dealloyed materials increases with increasing Cu content in the precursors, regardless of their crystal structure. For example, in the case of the Mg–Cu–Y alloys,^160^ a direct and linear relationship was found between the precursor’s Cu content (when between 20 and 40 at %) and the ligament size of the dealloyed morphology. With a few exceptions, this direct relationship between the Cu content in the precursor and the ligament/pore size of the np-Cu has also been reported in chemical dealloying of Ti–Cu alloys in various concentrations of HF at different temperatures.^145,146^ This trend can be attributed to the increase in the energy barrier for the dissolution of the LNE because of the increase in the concentration of the MNE in the precursor, which, in turn, promotes the surface redistribution of the MNE and consequently coarsening of the nanoporosity. Another contributing factor could be the higher oxygen affinity of the LNE than Cu, facilitating the surface diffusion of Cu and coarsening in precursors with higher Cu content, since there are fewer barriers on the surface originating from the oxidation of the residual LNE.

Cost optimization of the composition of the starting alloy requires using the least amount of the more expensive alloying element, which could be the MNE. Forming nanoporosity from lean noble alloys is often accompanied by crack formation,^173^ which may not be desirable depending on the intended application. The contributing factors in the cracking of the dealloyed layer include the stress relief from the removal of the LNE (similar to mudcrack formation in deserts after rainwater evaporation), the disproportionate extent of corrosion along the grain boundaries and consequent degradation of the mechanical properties, and coherency stresses caused by the lattice mismatch between the remaining MNE and the non- or less dealloyed backbone of the ligaments.

Precursor composition has been manipulated to reduce mudcrack formation and consequently improve the mechanical properties of the resulting nanoporous materials after dealloying. Wang et al.^167^ reported the suppression of large mudcracks found after electrochemical dealloying of Mn_70_Cu_30_ alloy (Figure 6a) by minor alloying it with elemental Si. The np-Cu samples resulting from the electrochemical dealloying of γ-Mn_70_Cu_28_Si_2_ showed significantly fewer and smaller mudcracks (Figure 6b). Wang et al. reported that a thin layer of Si-enriched “glue structure” with around 50 nm thickness was formed continuously along the grain boundaries (Figure 6b inset) and played the role of reinforcement for the dealloyed structure.^167^

The morphology of the nanoporosity is heavily influenced by the redistribution of the MNE during and after dissolution of the LNE. The ligament/pore size can be fine-tuned by alloying the precursors with elements with low surface diffusivity.^171,187^ A well-known example of reducing the length scale of nanoporosity with this technique is the alloying of Ag–Au systems with 1 to 3 at % of Pt.^173,188^ Aburada et al.^162^ explored the dealloying of Al-Cu-Mg-based amorphous alloys and the effect of a minor amount of Ni in the precursor alloy. A crystalline, Cu-rich, nanoporous structure with a pore diameter of 10 to 30 nm was formed as a result of the selective dissolution of Al and Mg and the redistribution of the remaining Cu. The nanoporous structure is smaller (around 10 nm) in the alloy containing Ni. The presence of Ni suppresses the surface diffusion of Cu. The decrease in surface diffusion, in turn, suppresses the coarsening of the ligaments in the already dealloyed layer (also referred to as postporosity coarsening), enabling tunable porosity development.

The use of preporous precursors for dealloying facilitates rapid nanoporosity evolution throughout the dimensions of the samples and shortens the total dealloying time and consequently mitigates postporosity coarsening, which refers to the continued coarsening of the dealloyed region as the dissolution penetrates deeper into the precursor. Depending on the size of the existing pores in the precursor, hierarchical porosity can be achieved and designed to obtain superhydrophobicity attributes for the dealloyed samples, as well as enhanced photocatalytic efficiencies. Yu et al. obtained preporous Al_67_Cu_33_ precursors via powder metallurgy by adding 20 weight (wt) % NaCl fine particles (around 70 μm) to the precursors’ powder mixtures, which were then sintered at 500 °C for 30 min, followed by a desalination process where the alloys were treated in a distilled water bath at 90 °C for 3 h.^133^ One of the most conspicuous unique features of np-Cu developed from Al–Cu foams is their hierarchical morphology, where length scale features of μm-level and nm-level are present that, in turn, increase their photocatalytic efficiencies for methyl orange degradation. Additionally, selective laser melting^14^ and direct-ink-writing based three-dimensional (3D) printing^189^ have been employed to fabricate preporous precursors and successfully resulted in nanoporous Cu structures with hierarchical morphology.

### 2.2 Effect of Phases and Crystal Structure of the Precursor Alloy

Obtaining homogeneous nanoporous layers is facilitated by using single-phase precursors, such as solid solutions, intermetallic compounds, and amorphous structures, whereas dealloying of multiphase precursors is more likely to result in a heterogeneous morphology. However, the presence of nondealloyed or less-dealloyed secondary phases, which are often encountered after dealloying of multiphase precursors, may enhance the mechanical stability of the nanoporous structures.

In some cases, the presence of more than one phase ends up being the very reason that dealloying becomes possible. For instance, Mg–Cu alloys containing around 67 at % Cu is made of a single MgCu_2_ phase, which does not dealloy completely; dealloying stops at the margins of the material due to the absence of a percolating path.^159^ However, at lower Cu concentrations (e.g., 60 atom %), a Mg_2_Cu phase is also present, and it is this Mg-rich phase that selectively dissolves away and provides a percolating path for the dealloying of the MgCu_2_ phase to proceed.

As dealloying is more likely to advance along the grain boundaries due to their higher energy, the dealloying front (i.e., the interface between the dealloyed layer and the nondealloyed substrate) in the case of polycrystalline precursors is less uniform than that of amorphous precursors.^160^ Furthermore, comparison between cold-rolled and annealed Cu–Mn precursors has revealed that a higher density of grain boundaries and dislocations reduces the dealloying time and results in a coarser porous structure.^168^ The anisotropic behavior of the cold-rolled samples during dealloying was revealed by faster dealloying kinetics along the rolling direction.^168^ Furthermore, the macroscopic grain structure and orientation of the starting alloy have been found to withstand nanoporosity evolution despite a change in crystal structure from BCC in the Zn–Cu precursor to FCC in the Cu rich nanoporous layer.^139^ However, dealloying at high temperatures in molten salts has been shown to alter the grain orientation of the dealloyed layer.^26^

### 2.3 Effect of Dealloying Potential

Electrochemical dealloying adds an extra level of control over the morphology of metallic nanoporous materials by influencing the relative kinetics of the dissolution of the LNE, the surface redistribution of the MNE, and the specific adsorption of anions. The competition among these phenomena can be manipulated by varying the applied potential during dealloying to control the morphology and size scale of the porosity. Higher redistribution rate of the MNE coarsens the nanostructure (larger ligaments and pores), whereas the increase in the dissolution rate of the LNE could result in smaller porosity because the rapid advancement of the dealloying front into the depth of the bulk precursor alloy leaves a shorter time for the surface redistribution of the MNE, hence inhibiting the formation of thicker ligaments. Adsorption of anions, such as halides, usually increases the surface mobility, resulting in enhanced coarsening, except for hydroxide ions, which reduce surface diffusion and hinder coarsening.

The higher the applied potential, the faster the dissolution of the LNE. Similarly, the redistribution of the MNE is facilitated by applying a higher dealloying potential provided that stationary surface species, such as adsorbed hydroxides, are not formed at such conditions. If the redistribution mechanism is governed by surface diffusion assisted by solvation of the MNE by electrolyte’s anions, then a higher applied potential could increase and strengthen the solvation in the adsorbed state; therefore, weakening the metallic bonds and facilitating the surface diffusion. If the redistribution occurs by localized dissolution followed by redeposition of the MNE, a higher applied potential facilitates the dissolution step, but if set high enough, it could inhibit the redeposition step and consequently prevent nanoporosity evolution since both elements would dissolve simultaneously. Therefore, the effect of the dealloying potential on the morphology is governed by the competition among the dissolution of the LNE, the surface redistribution of the MNE, and the effect of adsorbed species.

Experimental evidence, for instance, shows that a 50 mV increase (from 0.5 to 0.55 V vs mercury/mercury sulfate electrode (*V*_MSE_)) in the applied anodic potential during dealloying of Ag–Au–(Pt) alloys results in a smaller nanoporosity length scale,^188^ which is attributed to hydroxide adsorption (AuOH_ads_) hindering the surface mobility of Au. Conversely, Zheng et al.^140^ reported top surface SEM images showing larger nanoporosity when the applied potential during dealloying of Mn–Cu alloys was raised to a potential slightly higher than the thermodynamic prediction for Cu oxidation in acidic media,^190^ indicating that enhanced surface redistribution of Cu dominates the kinetics of Mn dissolution. This contrast between the effect of increased applied anodic potential on the morphology of the dealloyed Ag–Au-(Pt) alloys vs Mn–Cu alloys is related to the difference in the mechanism by which the MNE is redistributed at the dealloying interface. The proximity of the dealloying potential used in the case of Mn–Cu alloys^140^ to the equilibrium potential of Cu^2+^/Cu changes its redistribution mechanism from surface diffusion facilitated by solvation or anion adsorption (as is the case for Au) to a highly localized dissolution-redeposition mechanism.

### 2.4 Effect of Dealloying Electrolyte

Like the effect of the applied electrochemical potential, the electrolyte can also simultaneously influence the dissolution rate of the LNE and the redistribution rate of the MNE. The ability of the anions in the electrolyte to form soluble compounds with the cations of the LNE is crucial in nanoporosity evolution. Moreover, the affinity of the anions in the electrolyte toward the MNE leads to partial solvation and weakening of the metallic bonds, consequently facilitating the surface redistribution,^191^ or, as in the case of hydroxide adsorption inhibit surface diffusion of the MNE. The electrolytes used in the dealloying of Cu alloys that resulted in np-Cu structures include aqueous solutions such as H_2_SO_4_,^140,141,160,166^ HCl,^13,128,141,143,155,156,159,162,167^ HF,^145,146,163^ NaOH,^128,153,154^ NaCl,^165,168,172^ (NH_4_)_2_SO_4_,^141^ deep eutectic solvents such as choline chloride-urea,^169^ and ionic liquids such as 1-ethyl-3-methylimidazolium chloride.^174^

The use of HCl as an electrolyte has been shown to lead to a coarser ligaments from Al–Cu precursors compared to the structures formed in NaOH electrolytes.^155^ The presence of halides such as Cl^–^ ions in the electrolyte, which can form various complexes with Cu^+,^ for instance,^192,193^ accelerates the surface diffusion of Cu^191^ and induces significant coarsening as compared to electrolytes containing OH^–^. Lee et al.^154^ evaluated the effect of NaOH concentration (from 0.01 to 1 M) on the average ligament sizes and the composition of the dealloyed layer of Al_70_Cu_30_ precursors. The average ligament sizes of np-Cu films varied slightly (from 25 to 32 nm), with the coarser ligaments belonging to the samples dealloyed in more concentrated solutions. A significant amount of Al (up to 20 atom %) was reported to remain in the np-Cu films as the concentration of NaOH decreased to 0.01 M.

Concentrated acid media have been found to be detrimental to nanoporosity evolution in Cu alloys due to the solubility of Cu in certain strong acids. Chen et al.^13^ reported the formation of np-Cu by electrochemically dealloying the single-phase Mn_70_Cu_30_ alloy in HCl solutions of various concentrations. This study indicates that np-Cu is unstable in strong acid solutions, consistent with the literature.^145,146^ The optimal HCl solution concentration for the formation of uniform nanoporosity was found to be around 0.025 M.^13^

To reduce the feature size of np-Cu, surfactants have been employed to hinder the surface diffusion of Cu. Zheng et al.^140^ added sodium dodecyl benzene sulfonate (SDBS) surfactants to the electrolyte during chemical dealloying of Mn–Cu alloys in 0.075 M H_2_SO_4_ for 17 h and reported monolithic np-Cu with pore sizes from 5 to 15 nm as opposed to 50 ± 20 nm in the absence of the surfactant.

The active-passive behavior of the metals in the electrolyte is another important factor in selecting the dealloying media. For instance, nanoporosity evolution from dealloying Ti–Cu alloys has been achieved in HF electrolyte, whereas in 1 M HCl or 1 M HNO_3,_ only pitting corrosion has been reported.^146^ X-ray photoelectron spectroscopy analysis of the pitted surface has revealed the presence of Ti_2_O. The solubility of Ti_2_O in HF enables dealloying to proceed and nanoporosity to evolve. The influence of HF concentration on the pore and ligament sizes of dealloyed Ti–Cu alloys was also studied by Dan et al.^145,146^ A pore size of 25–75 nm and a ligament size of 46–79 nm were observed after dealloying Ti–Cu alloys of various compositions in 0.03 M HF solution at 298 K. In a 0.13 M HF solution, pore sizes increased (ranging between 85 and 380 nm), and so did the ligament sizes (ranging between 80 and 338 nm). The larger nanoporosity produced in the more concentrated electrolyte can be attributed to the secondary dealloying of the ligaments, as well as the increased mobility of Cu atoms at the solid/electrolyte interface as a result of more interactions between Cu and the anions in the electrolyte,^13^ further coarsening the structure.

Additionally, the passivity of the LNE in certain media has been used to dissolve the MNE and produce a nanoporous structure covered by the passive oxide layer of the LNE. Nanoporous Ni, for instance, has been developed from the dealloying of Ni–Cu alloys.^194^ The precursor alloy in this study was prepared by a prior electrodeposition step. Dealloying was performed at 0.5 V vs Ag/AgCl (3 M NaCl) for 3 h in the same solution as the one used for the electrodeposition step, that is, a pH 2.5 buffer solution of H_3_BO_3_ containing 1.6 M Ni­(H_2_NSO_3_)_2_ and 0.1 M CuSO_4_.

Moreover, the large potential window and the high boiling point of nonaqueous electrolytes, such as molten salts,^26^ ionic liquids,^174^ and deep eutectic solvents,^169^ enable the selection of the dealloying potential and temperature from a wider domain. Dealloying at elevated temperatures (in the range of 50–120 °C,^174^ for example) facilitates the evolution of crack-free^195^ and smooth nanoporous structures with relatively larger ligament-pore sizes.

### 2.5 Effect of Dealloying Temperature

Similar to the applied potential and the electrolyte used for dealloying, temperature also influences the kinetics of the dissolution of the LNE, as well as the redistribution of the MNE, both of which can be accelerated by dealloying at higher temperatures. Coarser nanoporous structures have been reported in several alloy systems, including noble alloys^188^ and Cu alloys^145^ when dealloying has been performed at elevated temperatures, indicating that the enhanced redistribution of the MNE, due to the increase in the surface diffusivity according to Arrhenius eq 1, dominates the kinetics of the dissolution of the LNE at higher temperatures.

$$D_{s}=D\cdot e^{-E_{a}/RT}$$ 1

In eq 1, *D*_0_ and *E*_a_ are the pre-exponential factor and the activation energy for adatom mobility, respectively, both of which are dependent on the nature of the materials involved, and *R* is the gas constant.

For example, Dan et al.^145^ dealloyed Ti–Cu amorphous alloys via free corrosion in 0.13 M HF for 3 h at different temperatures. Estimating the pore and ligament sizes of the nanoporous structure by the single chord length method over 150 sites of SEM images, they reported 34% and more than 100% coarsening of the nanoporosity for Ti_60_Cu_40_ and Ti_40_Cu_60_, respectively, as a result of increasing the dealloying temperature from 323 to 348 K. Moreover, Luo et al.^160^ studied the influence of dealloying temperature on the morphology of the np-Cu obtained from a Mg_65_Cu_25_Y_10_ amorphous precursor by chemical dealloying in 0.04 M H_2_SO_4_ at various temperatures ranging from 298 to 363 K for 20 min. A significant dependence was reported between the ligament size and the dealloying temperature; ligament size was found to increase more than 3 times (from ca. 48 to 158 nm) when the temperature was raised from 298 to 363 K.

### 2.6 Effect of Dealloying Time

The feature size of nanoporous materials can be tailored within a wide range by prolonging or curtailing the dealloying time, which influences the extent of postporosity coarsening.

Empirical evidence indicates a direct relationship between the coarsening of the ligaments and the dealloying duration, insofar as the MNE is stable in the dealloying media. For instance, Luo et al.^160^ investigated the effect of chemical dealloying time (10, 30, 90, and 300 min) on the microstructure stability of the np-Cu obtained from a Mg_65_Cu_25_Y_10_ amorphous precursor at room temperature (298 K) in H_2_SO_4_ solution. With the prolongation of leaching time from 1.5 to 5 h, for example, the results showed that the ligaments can be significantly coarsened (more than 2 times, from 80 to 200 nm). A similar trend was found in Lee’s work,^154^ where np-Cu was synthesized by dealloying Cu–Al precursor films in 0.5 M NaOH solution for different times. As a result of extending the dealloying time from 5 to 60 min, for instance, the average ligament size was found to increase by 84, 56, and 31% for Al_78_Cu_22_, Al_70_Cu_30_, and Al_63_Cu_37_, respectively. In other investigations, Dan et al.,^145,146^ studied the influence of chemical dealloying time on the morphology of nanoporous Cu from Ti_50_Cu_50_ precursors. With increasing immersion time from 10 min to 3 h, the length scales of the ligaments increased from several tens of nanometers to hundreds of nanometers in a 0.133 M HF solution.

The effect of postporosity coarsening of metallic nanoporous materials can be modeled with the Ostwald ripening theory^196^ and estimated using eq 2, which was used by Kaganovskiy and Ratinov in 1971 to measure the heterogeneous surface diffusion coefficient (*D*_s_) of Ag particles on Ni surface at high temperatures (700–900 °C):^197,198^

$${\mathrm{R̅}}^{4}=\frac{2\gamma a^{4}}{kT}D_{s}$$ 2

where *R̅* is the average radius of the coalescing particles at time *t*, *k* is the Boltzmann constant (1.380649 × 10^–23^ J/K), *T* is the absolute temperature, γ is the average surface energy (1.79 J/m^2^ for Cu^199^), *a* is the lattice parameter (3.6146 × 10^–10^ m for Cu^200^).

The average ligament size of nanoporous structures can be regarded as *R̅* in eq 2. Microscopy or diffraction measurements can be performed to monitor *R̅* over time and calculate *D*_s_ for different systems.

Additionally, to facilitate *D*_s_ estimation in aqueous media, in situ electrochemical measurements can be done to find the electrochemically active surface area and use its ratio to the geometric surface area, i.e., the roughness factor (*R*), which is known to have the following relationship with time.^201^

$$\frac{1}{R}\propto t^{1/4}$$ 3

However, in experiments, deviation from the above-mentioned formulations can occur due to several factors, including anion adsorption, retained LNE on the surface, and the presence of ternary elements with lower surface diffusivity. For the np-Cu system for example, Chen et al.^13^ reported that the average nanopore size of dealloyed Mn–Cu systems exhibits significant time dependence and increases with dealloying time, not according to eq 3, but near linearly, which is likely due to the retained Mn effect. The average nanopore size was found to be tunable from ∼15 to ∼120 nm by dealloying Mn_70_Cu_30_ in 0.025 M HCl under various dealloying times from 0.5 to 32 h, respectively.

## 3 Synthesizing Nanoporous Cu via Electrodeposition

Electrodeposition is a simple and inexpensive method for fabricating 3D porous metals and has been successful in developing np-Cu,^202^ np-Au,^203,204^ np-Ag,^205^ np-Pd,^203^ np-Sn,^206^ and np-Ni.^207,208^ Electrodeposition can produce a large array of morphologies by tuning the process parameters.^209^ Nanoporous structures fabricated by electrodeposition are well-suited for various applications in batteries,^210^ supercapacitors,^208^ fuel cells,^211^ and sensors.^10^ A metallic substrate of high electronic conductivity is typically used for electrodeposition, which constitutes an advantage for the fabrication of supercapacitor electrodes since the active material is directly applied on the current collector.^212^

In one of the reports of producing nanoscale Cu structures via electrodeposition, Shin and Liu^206,209^ developed a variety of np-Cu structures by taking advantage of hydrogen evolution to induce porosity. The pore sizes and ligament structures of the foams are tunable by adjustment of the deposition conditions. The size of the pores in a 100 μm-thick foam was reduced from 50 to about 25 μm by adding 0.1 M acetic acid to the electrolyte of 0.4 M CuSO_4_ and 1.5 M H_2_SO_4_. With the addition of 1–50 mM HCl, the average size of the elementary branches in the foam wall was reduced from 300 to 50 nm, forming nanostructured porous electrodes that could be used in electrocatalytic applications.

More research on macro- and mesoporous Cu by electrodeposition can be seen in Table 3.

**Table 3:** Summary of Porous Cu Structures Developed via Electrodeposition

working electrode (cathode)	counter electrode (anode)	applied cathodic current density (A cm^–2^)	*T* (K)	deposition time	electrolyte	morphology	ref
99.8% Cu	Cu plates	–3	RT^a^	5 s	1.5 M H_2_SO_4_ + 0.02 M CuSO_4_	the pore size of the 3D microfoam structure increased with the time of deposition; more importantly, the walls of the microfoam are also highly porous due to vigorous hydrogen evolution originating not only at the substrate but also at the deposited copper dendrites	^206^
				10 s			
				20 s			
99.8% Cu	Pt electrode	–3	RT^a^	10 s	0.03–0.5 M CH_3_COOH + 0.2–0.8 M CuSO_4_ + 0.1–1.5 M H_2_SO_4_ + 1–50 mM HCl (as a catalyst)	the size of the surface pore of a 100 μm-thick foam was reduced from 50 to about 25 μm by adding 0.1 M acetic acid to the deposition bath; with the addition of 1–50 mM HCl, the size of the Cu branches was dramatically reduced; in particular, the average size of the elementary branches in the foam wall was reduced from 300 to 50 nm	^209^
				20 s			
				40 s			
				60 s			
Cu sheet	Pt wire	–1.3	RT^a^	28 s	0.4 M CuSO_4_ + 1.5 M H_2_SO_4_	the fabricated foam exhibits a μm-sized porous structure; the ligaments of fabricated foam are composed of numerous small-ramified deposits	^202^
pure Cu plate	a Cu plate containing phosphorus	–0.01	298	IIM^b^	0.85 M CuSO_4_ + 0.55 M H_2_SO_4_ + (C_3_H_4_O_2_)*_n_* (mean molecular weight 5000; PA-5000)	a 3D copper nanostructured architecture that consists of sheet-like copper deposits with a thickness of 50 nm and relatively high porosity was fabricated by the addition of PA-5000	^213^
Au (200 nm thick) electron beam deposited on glass Cu foil C paper activated by immersing in concentrated HNO_3_ for 1 hC paper sputter-coated with Cu (∼0.01 mg cm^–2^)	Pt wire	ranging from −0.008 to −0.001	IIM^b^	500 s	0.1 M CuSO_4_ + H_2_SO_4_ (pH ranging from 1 to 3) + 10 mM of one of the following additives: DAT,^c^ DTAB^d^ or ThonB^e^	in the presence of the additives, the deposition of Cu was inhibited; the strongest inhibition was reported for ThonB, followed by DTAB and DAT	^72^
						Cu deposition inhibition by DAT was seen to increase with increasing pH; at higher pH, Cu-DAT complexes were detected with UV–vis spectroscopy; reproducible and uniform Cu deposition was not achieved at pH higher than 3	
						H_2_ evolution could not be detected in the presence of the additives	
						the morphology of the deposited Cu was smooth and uniform in the absence of the additives, as well as in the presence of ThonB and DATB, the two of which performed as levelers	
						nanoporous morphology was obtained only in the presence of DAT	
						varying the pH from 1 to 1.5 to 2.5 in the presence of DAT, the Cu depositions exhibited dot shape, wirelike, and ill-defined morphologies, respectively	
						higher deposition current density increased the nucleation density of Cu, resulting in smaller-sized Cu nanostructures	
						no particular difference was detected among depositions on different substrates	
Au (200 nm thick) electron beamC paper activated by immersing in concentrated HNO_3_ for 1 h	Pt wire	4 mA/cm^2^	IIM^b^	until a final deposition charge of 2 C/cm^2^	0.1 M CuSO_4_·5H_2_O + 10 mM DAT,^c^ with or without 1 mM Ag_2_SO_4_, at pH = 1.5 adjusted by using H_2_SO_4_	electrodeposition in the electrolyte containing DAT^c^ but without Ag salt resulted in a wire-like morphology	^69^
						the absence of DAT^c^ and the presence of Ag salt in the electrolyte resulted in large particles on the surface	
						in the presence of both DAT^c^ and the Ag salt, substantial porosity was obtained with wire-like morphology containing 6% Ag	
high-purity Cu	Pt electrode	–3	RT^a^	10 s	CuSO_4_ (0.2–0.8 M) + 1.5 M H_2_SO_4_ + CH_3_COOH (0.03–0.2 M) as bubble stabilizer	a three-dimensional copper foam made up of multiple nanostructure dendritic ligaments was created and showed interconnected pores ranging from 20 to 50 μm; the size of the branches of copper was less than 1 μm; higher CuSO_4_ concentrations resulted in smaller pores; the presence of acetic acid suppressed the combination of H_2_ bubbles, resulting in a higher level of porosity	^214^
				20 s			
				40 s			
				60 s			
Au	Pt mesh electrode	–0.1	293 ± 1	45 s	0.04, 0.06, 0.08, 0.1 M CuSO_4_ + 0.1, 0.5 M H_2_SO_4_ + 10 μM −5 mM CTAB^f^	the pore diameters and wall thickness of the porous copper films were successfully tailored by adjusting the concentration of the electrodeposition electrolyte, the applied current density, and the concentration of the surfactant (CTAB)	^215^
		–0.2		85 s		as the concentration of CuSO_4_ was increased from 0.04 to 0.08 M, the pore size increased from 40 to 100 μm, and the wall thickness increased from 20 to 60 μm; at CuSO_4_ concentrations higher than 1.5 M porous structure was not obtained	
		–0.4				higher current densities (from 0.1 to 1.2 A cm^–2^) resulted in an increased rate of H_2_ evolution and formation of smaller bubbles, hence decreased pore size (from 150 to 50 μm); the wall thickness of the porous structure was reduced (from 65 to 25 μm) as the Cu deposition was expedited at higher current densities (from 0.1 to 1.2 A cm^–2^)	
		–0.8				up to 1 mM (critical micelle concentration), the increase in the concentration of the CTAB surfactant decreased the pore size and the wall thickness; by stabilizing the H_2_ bubbles and preventing their coalescence, a higher level of porosity can be achieved using surfactant	
		–1.2					
stainless-steel plate	Pt plate	–0.6	RT^a^	30 s	0.5 M NiSO_4_ + 1.5 M H_2_SO_4_ + 1 M HCl + 0.01 M CuSO_4_	for high current densities (−1.5 and −1.8 A cm^–2^), the nickel–copper deposits have a three-dimensional foam-like morphology with randomly distributed nearly circular pores (5–20 μm) whose ligaments present an open dendritic structure	^212^
		–1		60 s			
		–1.5		90 s			
		–1.8		180 s			

a Room temperature.

b Information is missing.

c 3,5-Diamino-1,2,4-triazole (DAT).

d Dodecyltrimethylammonium bromide (DTAB).

e Thonzonium bromide (ThonB, hexadecyl­[2-[(4-methoxyphenyl)-methylpyrimidin-2-ylamino]­ethyl]­dimethylazanium bromide).

f Cetyltrimethylammonium bromide (CTAB).

Most of the published work used a copper sheet/foil as substrates to synthesize np-Cu. However, Eugenio et al.^212^ attempted stainless steel substrates, due to its relative low cost, good conductivity, and high corrosion resistance, to electrodeposit Ni–Cu highly porous metallic foams using the dynamic hydrogen bubble template for supercapacitor applications. In their study, by controlling the applied cathodic current densities above 1 A cm^–2^, macroporous Ni–Cu metallic foams (pore and ligament sizes ranged from 5 to 20 μm) with randomly distributed nearly circular pores and ligaments that have open dendritic structures were obtained on stainless steel substrates. However, dendritic structures typically suffer from low mechanical stability, hence cannot be considered for long-term applications unless heat-treated, which sinters the dendrites into larger ligaments.

The stability of the H_2_ bubbles produced during the electrodeposition can be increased by the addition of surfactants, which adsorb on the gas and liquid interfaces and inhibit the collision and coalescence among them. The bubble size is then kept small, rendering a smaller porous structure after electrodeposition. Li et al.,^215^ for instance, synthesized macroporous copper films on Au substrates by electrodeposition in 0.5 M H_2_SO_4_ solutions containing various concentrations of CuSO_4_ and cetyltrimethylammonium bromide (CTAB) surfactant. The presence of CTAB surfactant (if kept under the critical micelle concentration, which was found to be around 1 mM) reduced the pore size and ligament thickness of the resulting macroporous Cu.

Moreover, in this study, Li et al. were able to obtain a superhydrophobic surface (a contact angle of larger than 150°) for Cu.^215^ They achieved a double roughness structure by controlling the deposition parameters and the electrolyte such that a hierarchical porous film with 10–150 μm pores composed of dendrites of approximately 30 nm was obtained.

Compared to dealloying, the main shortcomings of the electrodeposited Cu films in synthesizing highly porous morphologies are the often-larger size of porosity reached by this technique and the nonuniform distribution of the pore size in the cross-sectional direction (vertical to the substrate). These morphological characteristics are adverse to maximizing the surface area, as required for the enhancement of the applications in sensing, catalysis, supercapacitors, and batteries.

To synthesize uniform np-Cu, Liu et al.^210^ employed electrical field-induced assembly of Cu nanoparticles (NPs). They produced a Cu NP dispersion that is colloidally stable against oxidation and agglomeration within a time frame sufficient for the fabrication of porous Cu films, and simultaneously is capable of self-assembly in aqueous solutions. In this study, electrochemistry was only used to evolve H_2_ from a Cu foil, serving as a negative template for the coalescence of Cu NPs resulting in a np-Cu morphology. While this approach eliminates the considerations and limitations of dealloying, which necessitates at least a binary alloy of a MNE and a LNE, the synthesis of desired NPs may be challenging too. Liu et al.^216^ developed stable Cu NPs by stirring and ultrasonication of equal volumes of 0.06 M CuCl_2_ + 0.01 M CTAB and 0.8 M l-ascorbic acid + 0.01 M CTAB aqueous solutions then mixing the two and heating at 45 °C for 42 h under vigorous stirring. The size and shape of the Cu NPs can be controlled by changing the reaction time and composition of the precursor solutions.

## 4 Summary and Perspectives

Copper and its alloys can play a strategic role in the design and engineering of technologies that are crucial to a sustainable human society. By catalyzing the electrochemical conversion of CO_2_ into fuels and valuable chemicals, Cu-based electrodes can contribute to closing the carbon cycle and facilitate the sustainable production of energy carriers. Furthermore, the application of Cu in self-sanitizing surfaces offers sustainable mitigation strategies against infectious diseases and global pandemics.

The development of high roughness factor Cu-based materials can significantly enhance the catalytic and biocidal performance, as these are surface-based phenomena. Thus, synthesis methods such as dealloying and electrodeposition are not merely fabrication routes, but structure-directing strategies that determine how effectively Cu interfaces with reaction intermediates in CO_2_ reduction and with microbial and viral membranes in biocidal applications.

After introducing dealloying and electrodeposition techniques, which are among the most controllable, facile, and cost-effective methods for producing monolithic nanoporous metallic materials, the influence of various synthesis parameters that can be leveraged to fine-tune the nanoporous morphology and obtain the desired roughness factor was reviewed. For dealloying, the composition of the precursor alloy must be designed considering the percolation threshold and parting limit. The crystal structure or the lack thereof (as in amorphous precursors) affects the uniformity of the resulting nanoporous network. Parameters such as dealloying potential, electrolyte, temperature, and time jointly govern the dissolution of the less noble element and the redistribution of the more noble element, i.e., Cu. Generally, higher temperatures and longer treatment times favor coarser nanoporosity due to enhanced surface redistribution kinetics, while lower potentials and shorter times can lead to finer features. Understanding these relationships provides a foundation for rationally tailoring the nanoporous morphology toward specific functional outcomes in CO_2_ reduction and self-sanitizing applications.

However, before nanoporous Cu can transition to large-scale practical implementations, several challenges must be addressed to fully align the structure with function:

(I) Structural and operational stability: the high surface energy that endows np-Cu with exceptional reactivity also drives coarsening and morphology collapse during prolonged electrochemical or environmental exposure. Under CO_2_ reduction conditions, potential-induced restructuring alters the active site distribution and can shift selectivity away from multicarbon products. For biocidal applications, mechanical wear and repetitive wet–dry cycles can compromise the integrity of the porous network. Future efforts should focus on kinetic stabilization strategies, such as alloying with immobile elements, oxide-metal hybridization, or dynamic self-healing surface chemistries, to retain nanoscale architecture under realistic operating environments.
(II) Surface oxidation and dynamic phase evolution: the catalytic and antimicrobial behaviors of Cu are strongly coupled to its redox flexibility. Transitions between Cu^0^, Cu^+^, and Cu^2+^ species govern CO_2_ adsorption, CO intermediate stabilization, and formation of reactive oxygen species. However, these transitions remain poorly understood under dynamic potentials or ambient humidity. Real-time spectroscopic and microscopic studies are needed to map how oxide phases nucleate, dissolve, and reform under operando conditions, thereby enabling the rational tuning of redox-active surfaces that balance activity with durability.
(III) Morphological control and nanostructure–function correlation: while dealloying and electrodeposition allow fine-tuning of the pore size and ligament geometry, predictive control remains empirical. The interplay between local curvature, confinement effects, and charge distribution in nanoporous frameworks profoundly impacts both catalytic selectivity and ion release kinetics. Establishing quantitative nanostructure–function relationships through systematic experimental mapping and multiscale simulations will be key to designing morphologies that maximize the desired functionalities while minimizing parasitic reactions.
(IV) Hierarchical and multifunctional architectures: real-world devices demand structural sophistication beyond a single-scale porosity. Hierarchical frameworks combining macro-, meso-, and micropores could simultaneously enable efficient mass transport, bubble detachment, and wetting control in CO_2_ electrolysis, while providing increased surface area and controlled hydrophobicity for antimicrobial use. However, the scalable synthesis of such architectures with uniform pore connectivity and mechanical robustness remains a formidable challenge. Innovative templating, gradient dealloying, and additive manufacturing strategies may offer viable pathways forward.
(V) Integration and system-level performance: ultimately, the impact of np-Cu will depend on its integration into functional assemblies, such as gas diffusion electrodes, membranes, or surface coatings, where transport phenomena, interfacial adhesion, and cross-scale coupling govern performance. Standardized testing protocols and durability benchmarks across both electrocatalytic and antimicrobial contexts are essential to compare material designs and translate laboratory insights into deployable technologies.

Overall, advancing np-Cu toward catalytic and biocidal technologies requires coupling synthesis control with mechanistic insight. Future studies should focus on establishing quantitative correlations between the morphology and performance metrics under realistic operating conditions. In situ and operando characterization techniques, complemented by atomistic modeling, can bridge the gap between structural design and functional behavior. By unifying principles of materials synthesis, surface science, and electrochemical engineering, Cu-based nanoporous materials can be rationally designed to meet the challenges of CO_2_ recycling and the mitigation of pathogen transmission.
